# Phosphate deficiency induced by infection promotes synthesis of anthracnose-resistant anthocyanin-3*-O-*galactoside phytoalexins in the *Camellia sinensis* plant

**DOI:** 10.1093/hr/uhad222

**Published:** 2023-12-05

**Authors:** Tongtong Li, Shenrong Wang, Dandan Shi, Wen Fang, Ting Jiang, Lixin Zhang, Yajun Liu, Liping Gao, Tao Xia

**Affiliations:** State Key Laboratory of Tea Plant Biology and Utilization/Key Laboratory of Tea Biology and Tea Processing of Ministry of Agriculture/Anhui Provincial Laboratory of Tea Plant Biology and Utilization, Anhui Agricultural University, West 130 Changjiang Road, Hefei 230036 Anhui, China; School of Life Science, Anhui Agricultural University, Hefei 230036, Anhui, China; School of Life Science, Anhui Agricultural University, Hefei 230036, Anhui, China; School of Life Science, Anhui Agricultural University, Hefei 230036, Anhui, China; School of Life Science, Anhui Agricultural University, Hefei 230036, Anhui, China; Anhui Province Key Laboratory of Integrated Pest Management on Crops, College of Plant Protection, Anhui Agricultural University, Hefei 230036, China; School of Life Science, Anhui Agricultural University, Hefei 230036, Anhui, China; School of Life Science, Anhui Agricultural University, Hefei 230036, Anhui, China; State Key Laboratory of Tea Plant Biology and Utilization/Key Laboratory of Tea Biology and Tea Processing of Ministry of Agriculture/Anhui Provincial Laboratory of Tea Plant Biology and Utilization, Anhui Agricultural University, West 130 Changjiang Road, Hefei 230036 Anhui, China

## Abstract

Tea (*Camellia sinensis*) is a well-known beverage crop rich in polyphenols with health benefits for humans. Understanding how tea polyphenols participate in plant resistance is beneficial to breeding resistant varieties and uncovering the resistance mechanisms. Here, we report that a *Colletotrichum* infection-induced ‘pink ring’ symptom appeared outside the lesion, which is highly likely to occur in resistant cultivars. By identifying morphological feature-specific metabolites in the pink ring and their association with disease resistance, and analysis of the association between metabolite and gene expression, the study revealed that the accumulation of anthocyanin-3-*O*-galactosides, red phytotoxin compounds resistant to anthracnose, plays a pivotal role in the hypersensitive response surrounding infection sites in tea plants. The results of genetic manipulation showed that the expression of *CsF3Ha*, *CsANSa*, *CsUGT78A15*, *CsUGT75L43*, and *CsMYB113*, which are involved in anthocyanin biosynthesis, is positively correlated with anthracnose-resistance and the formation of the pink ring. Further phosphorus quantification and fertilization experiments confirmed that phosphate deficiency caused by anthracnose is involved in the occurrence of the pink ring. Genetic manipulation studies indicated that altering the expression levels of Pi transporter proteins (*CsPHT2-1*, *CsPHT4;4*) and phosphate deprivation response transcription factors (*CsWRKY75-1*, *CsWRKY75-2*, *CsMYB62-1*) enhances resistance to anthracnose and the formation of the pink ring symptom in tea plants. This article provides the first evidence that anthocyanin-3-*O*-galactosides are the anthracnose-resistant phytoalexins among various polyphenols in tea plants, and this presents an approach for identifying resistance genes in tea plants, where genetic transformation is challenging.

## Introduction

Tea beverage, made from young tea leaves rich in tea polyphenols, is one of the three most popular non-alcoholic beverages (cocoa, coffee, and tea) in the world [1–3]. Tea polyphenols with multiple phenolic hydroxyl groups have attracted scientists' attention due to their antioxidant characteristics [4]. According to their structural characteristics, polyphenol compounds from tea plants (*Camellia sinensis*) can be divided into phenolic acids, flavan-3-ol monomers and polymers, flavonols and flavonoids, and anthocyanins [5, 6]. Their metabolic pathways in tea plants, involving the shikimic acid, phenylpropane, flavonoid, and galloyl derivative pathways, are clearly understood [7–12]. Furthermore, transcription factors responsible for the regulation of flavonoid pathways, such as CsMYB5s and CsMYB75, have been identified in tea plants [13–16].

Due to the antioxidant ability of phenolic hydroxyl groups, different types of tea polyphenol compounds show good antibacterial ability in *in vitro* antimicrobial tests [17]. However, the role of phenolic compounds in the immune system of tea plants is still not well understood. There is no direct evidence from *in vivo* tests about whether tea plants can resist pathogenic microorganisms via the immune system. Some research has showed that tea polyphenols participate in resistance to biological and abiotic stresses in tea plants, such as insects and pathogenic bacteria [18, 19]. Identification of key resistance genes or mechanisms in tea plants is a difficult task for researchers due to the absence of an efficient genetic transformation protocol.

Anthracnose is a worldwide plant disease caused by *Colletotrichum* spp. and is prevalent in all tea regions of China, particularly in warm and humid tropical and subtropical areas [20]. Studies have shown that certain species of *Colletotrichum* are responsible for the production of anthracnose in tea plants, including *C. camelliae*, *C. fructicola*, *C. aenigma*, *C. endophytica*, *C. siamense*, and *C. karstii* [21, 22].

Plant defense compounds are a class of compounds produced when plants are attacked by pathogens. They are divided into two types: phytoalexins and phytoanticipins. Phytoalexins are low molecular weight antimicrobial compounds that do not pre-present in plants but are synthesized *de novo* after infection by pathogens. Phytoanticipins are low molecular weight antibacterial compounds originally existing in plants or synthesized after infection by pathogens from pre-existing plant constituents [23]. In fact, these two kinds of compounds cannot be clearly distinguished. The difference between phytoalexins and phytoanticipins lies in their biosynthesis context.

At present, many kinds of compounds have been identified as phytoalexins or phytoanticipins, including indoles, isoprenoids, flavonoids/isoflavonoids, benzophenanthridine alkaloids, pterocarpans, wyerone epoxide, and *o*-hibiscanone [24, 25]. Camalexin, a sulfur-containing indole alkaloid, is the most important phytoalexin in *Arabidopsis* to resist pathogens. Its synthesis is regulated by ethylene, jasmonic acid (JA), and mitogen-activated protein kinase (MAPK) signaling pathways [26]. Many phytoalexins belong to the phenolic compounds, which are derived from phenylpropane and flavonoid synthesis pathways, such as the most well-known polyphenolic stilbenoid, resveratrol, present in grapes, mulberries, peanuts, and rhubarb [23]. Isoflavonoid phytoalexin glyceollins can reduce the oxidative damage induced by fungal pathogens and oomycetes in leguminous plants [27]. 3′-Deoxyanthocyanidin, a phytoalexin in sorghum, is synthesized *de novo* via the flavonoid biosynthesis pathway after anthracnose induction [28, 29].

**Figure 1 Appearance of:**
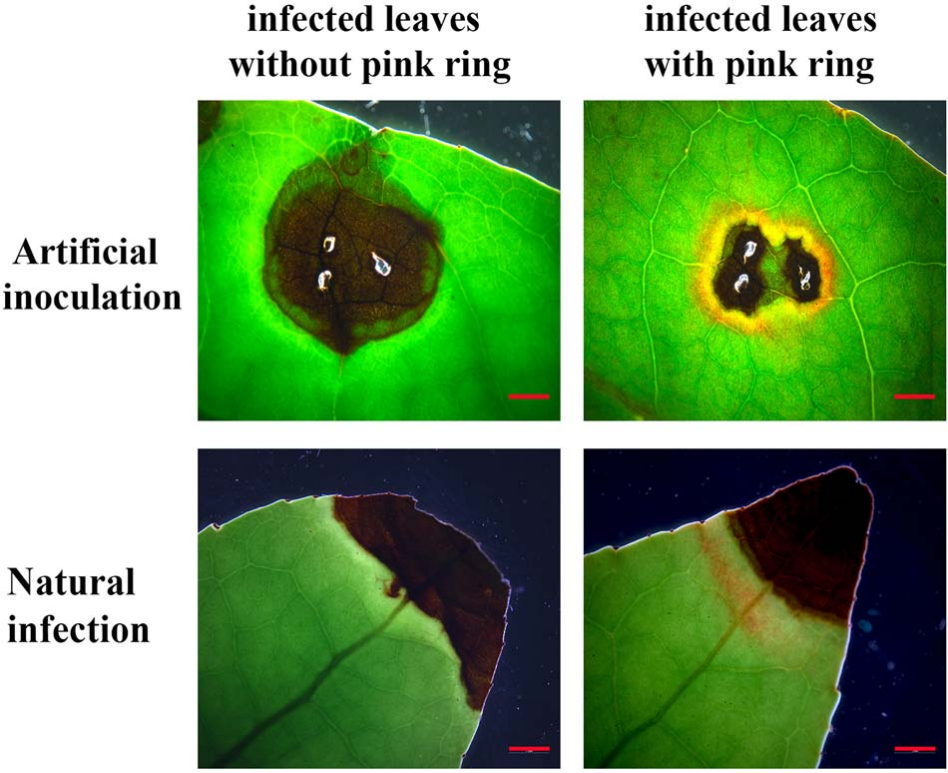
the pink ring around the lesion in tea plants infected with *C. camelliae*. Images of lesions were obtained from artificially inoculated and naturally infected tea leaves. Two different phenomena were observed in the infected tea leaves: infected leaves with pink rings (right) and infected leaves without pink rings (left). Scale bar = 2 mm.

Research has shown that after tea leaves are infected with *Colletotrichum* spp*.*, small brown concentric spots appear on the leaves first and then gradually develop into large round or irregular brown or black lesions [22]. In this article, an inconspicuous phenomenon was observed: when the leaves of healthy tea seedlings were inoculated with *C. camelliae*, a ring consisting of a pink isolation belt often appeared outside the lesion of the infected part. It was named *Colletotrichum* infection-induced ‘pink ring’. Atypical pink ring symptoms were also seen on leaves naturally infected with *C. camelliae* in the greenhouse (Fig. 1). This study focuses on the changes in gene expression levels during the infection process of *C. camelliae*, as well as the identification of anthracnose-resistant phytoalexins specifically accumulated in the pink ring. It investigates the regulatory mechanisms underlying the formation of these phytoalexins and their role in resisting invasion by *Colletotrichum acutatum*.

## Results

### A pathogenic isolate of *C. camelliae* infection-induced pink ring symptom and its cellularity

**Figure 2:**
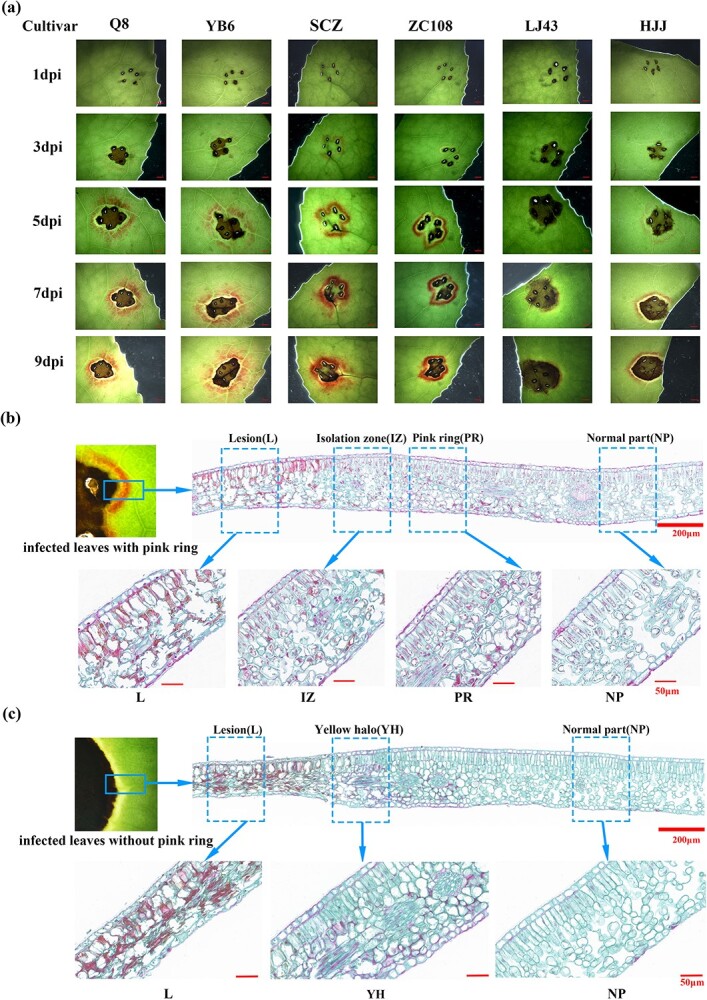
Pink ring symptoms on inoculated leaves of different tea cultivars and paraffin sectioning of pink ring tissue. **a** Pink ring symptom on leaves of different tea cultivars within 9 dpi. Scale bar = 2 mm. **b**, **c** Observation of cell morphology of lesion extending into healthy leaf areas using paraffin sectioning.

To observe whether the pink ring exists universally in tea plants infected by anthracnose, several tea cultivars were used for the infection test. The leaves of healthy 2-year-old seedlings of each cultivar were inoculated with *C. camelliae* to induce the occurrence of anthracnose (Fig. 2a). Within 9 days post-inoculation (dpi), the lesions appeared around the inoculation point of leaves and slowly expanded. Under a stereo microscope, a red halo was observed around the lesion on the upper side of infected leaves at 5 dpi. There was an isolation zone between the red halo and lesion, and its leaf color was still green. We described this red halo as a pink ring. In addition, it was reported that cultivar ‘Zhongcha108’ (ZC108) had higher resistance to *Colletotrichum* than the cultivar ‘Longjing43’ (LJ43) [30]. We also found that the frequency of the pink ring on the infected leaves of the susceptible cultivar LJ43 was lower than that of the resistant cultivar ZC108.

It is reported in the literature that 3′-deoxyanthocyanidin, as a phytoalexin, appears in the inclusions of host epidermal cells and is released to prevent the spread of the pathogenic fungus [29, 31]. To determine the cellularity of the pink ring, a series of hand and paraffin sections of infected leaves were prepared (Fig. 2b, Supplementary Data Fig. S1a). The results showed that red compounds mainly accumulated in the columnar palisade cells of the mesophyll tissue of the pink ring (Supplementary Data Fig. S1a). In addition, a small amount of intact protoplasts containing red compounds could be isolated and purified from the position of the pink ring, indicating that red compounds are located in vacuoles or vesicles.

The paraffin sections further clearly revealed the cell structural characteristics of the infected leaves. In leaves with the pink ring there were four parts, comprising the normal part, the lesion, the pink ring, and an isolation zone between the lesion and the pink ring (Fig. 2b). In infected leaves without a pink ring there were three parts, comprising the normal part, the lesion, and a yellow halo (Fig. 2c). The chloroplasts and vacuoles were arranged regularly in the cells of the palisade and spongy mesophyll tissues, and the cell gaps of the spongy mesophyll tissue were clearly visible in the normal parts of the two types of infected leaves mentioned above. In comparison, no complete cell structure was seen in the lesions of the two types of infected leaves mentioned above; chloroplasts disappeared, accompanied by a huge cavity present in the palisade and spongy mesophyll tissues, indicating that the cells had died. The cell contours of palisade and sponge tissues in the isolation zone and the phenomenon of plasmolysis were visible, suggesting that the cells were still living. However, the gap between cells in this area was reduced, cell volume had significantly increased, and chloroplast structure was blurry, indicating that the cells were undergoing necrosis. There was no significant difference between the cells in the pink ring and the isolation zone, but the cell structure, including the chloroplast structure, was more obvious in the pink ring. The cellular structure of the area with the yellow halo was almost identical to that of the area with the pink ring. It is speculated that the hypersensitive response and programmed cell death activation caused by anthrax infection occurred in the cells of these parts. In summary, the synthesis of the red compounds induced by anthrax infection may be a part of the hypersensitive response of cells around infection sites in tea plants.

**Figure 3:**
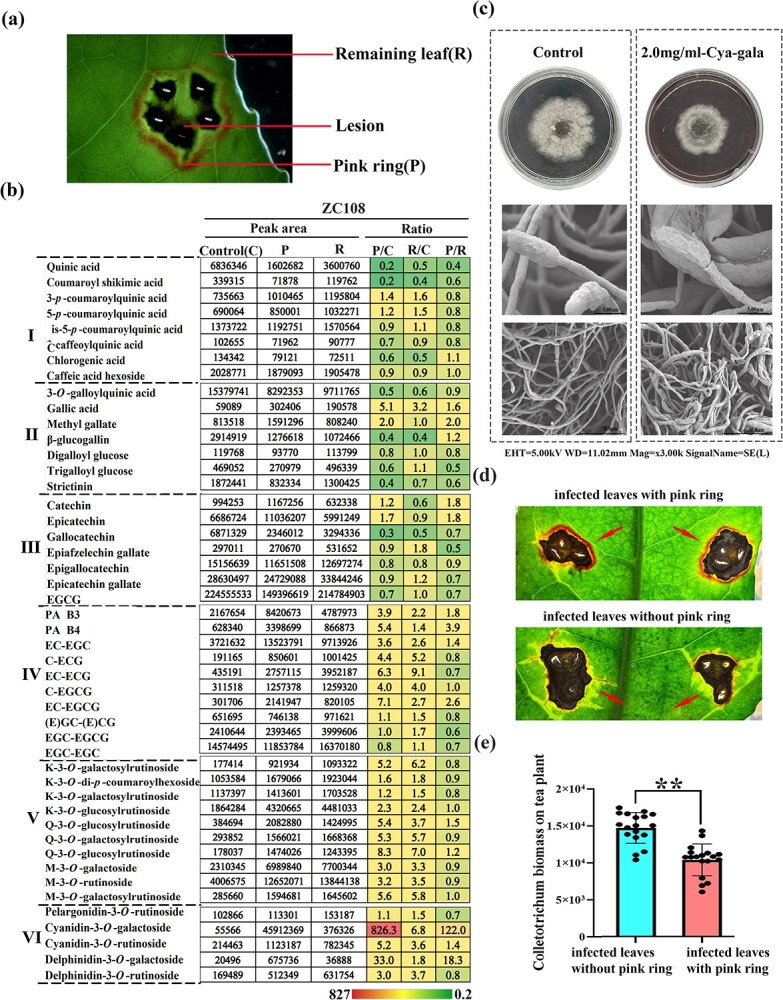
Detection of main phytoalexin compounds in the pink ring and their disease resistance. **a** Schematic diagram of the sampling site of metabolite detection. **b** Contents of phenolic metabolites located in the pink ring (P) and in the remaining leaf area (R) outside the pink ring of infected leaves of the resistant cultivar ‘Zhongcha108’ and the ratio of P to R. I, phenolic acids; II, gallic acid and its derivatives; III, flavan-3-ol monomers; IV, flavan-3-ol polymers; V, flavonol glycosides; VI, anthocyanins. **c** Inhibition by cyanidin-3*-O-*galactoside of growth of the pathogenic fungus *C. camelliae in vitro*. Electron micrograph of *C. camelliae* under cyanidin-3*-O-*galactoside treatment. **d** Observation of the spread of lesions in infected leaves with pink rings and infected leaves without pink rings 6 days after *C. camelliae* infection. **e** Disease index of *C. camelliae*-injected plants at 6 dpi. Samples in (**d**) were subjected to a pathogenic fungicidal recovery assay. Leaves around the lesion were sterilized and placed on PDA medium and incubated at 25°C. Photographs were taken, and mycelial biomass statistics were determined after 2 days of incubation. Error bars represent ± standard deviation (*n* = 18), *P < 0.05, **P < 0.01.

### Identification of metabolites in the pink ring and their action in anthracnose resistance

Our published UPLC–QqQ–MS/MS (ultra-performance liquid chromatography–triple quadrupole mass spectrometry) technique was used to detect phenolic metabolites located in the pink ring and the remaining leaf area outside the pink ring of infected leaves [6] (Fig. 3a and b). Phenolic acids, flavan-3-ol monomers, proanthocyanidins (PAs), flavanol and flavone glycosides, and anthocyanin glycosides could be identified and quantified by using the charge mass ratio of their characteristic ions. The results showed that the relative contents of PAs, flavonol glycosides, and anthocyanin glycosides both in the pink ring and the remaining leaf area outside the pink ring were significantly higher than those in the control leaves without *C. camelliae* inoculation. This phenomenon indicated that the synthesis of these phenolic substances in the infected leaves, whether inside or outside the pink ring, was upregulated, even far from the infection point. Notably, the content of flavan-3-ol gallates, such as epigallocatechin gallate (EGCG), the main phenolic compound in tea leaves, was only 70% of that in the control. Similarly, the content of simple hydrolyzed tannins (such as strictinin) decreased in infected leaves. This means that galloylation, which is related to flavan-3-ol gallate and hydrolyzed tannin biosynthesis [11, 12], may not be upregulated in infected leaves. Among polyphenols, flavonol glycosides and PAs are more likely to be associated with systemic acquired resistance in tea plants.

Further comparative analysis showed that the content of anthocyanin glycosides in the pink ring increased significantly compared with the remaining leaf area. Among them, cyanidin-3*-O-*galactoside and delphinidin-3*-O-*galactoside contents were 122 and 18.32 times those in the leaf area outside the pink ring, respectively. However, the contents of their rutinosides did not increase significantly.

In addition, almost all flavonol glycosides (except for a few with two hydroxyl groups in the B ring) and flavone glycosides increased inside and outside the pink ring. However, their contents in the leaf area outside the pink ring were slightly higher than that in the pink ring, which was contrary to the change in anthocyanins in infected leaves.

The content of catechins or flavan-3-ol monomer compounds with B-ring dihydroxy groups, such as epicatechin and catechin and their polymers procyanidin B1, procyanidin B3, and procyanidin B4, increased more in the pink ring than in the remaining leaf area. In conclusion, anthocyanin glycosides are involved in localized acquired resistance in tea plants. Among them, cyanidin-3*-O-*galactoside is probably the main phytoalexin compound. The freehand sections of infected leaves showed the accumulation of anthocyanins in vacuole (Supplementary Data Fig. S1b).

To verify whether cyanidin-3*-O-*galactoside exhibited anti-anthracnose function, an *in vitro* test to inhibit the growth of *C. camelliae* was performed. The area of *C. camelliae* was significantly smaller than that of the control group on PDA medium containing 2 mg ml^−1^ cyanidin-3*-O-*galactoside. The results indicated that cyanidin-3*-O-*galactoside had an apparent inhibitory effect on the growth of *C. camelliae*. The mycelia of *C. camelliae* showed signs of shriveling, atrophy, and deformation under treatment with cyanidin-3*-O-*galactoside (Fig. 3c).

It could also be observed that the pink ring can inhibit the diffusion of *C. camelliae* on the infected tea leaves. Signs of the spread of the lesions could be seen at the edge of lesions of infected leaves without pink rings but not on the edge of infected leaves with pink rings (Fig. 3d). Subsequently, we performed mycelial recovery experiments around the lesions of leaves with or without pink rings (Supplementary Data Fig. S2). The quantitative detection of mycelium content showed that the amount of mycelium around the pink ring was significantly less than that around lesions without the pink ring (Fig. 3e). This indicated that the pink ring around the lesions was beneficial to controlling the spread of *C. camelliae.*

### Cultivar ‘Zijuan’, rich in anthocyanins, has higher resistance to anthracnose

**Figure 4:**
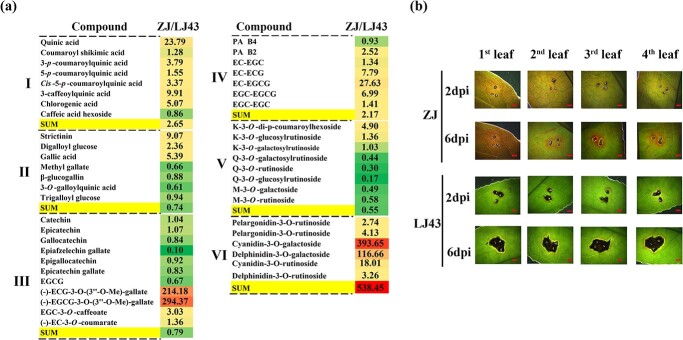
Cultivar ZJ, rich in anthocyanins, has higher resistance to anthracnose. **a** Comparative analysis of phenolic metabolite content between the resistant cultivar ZJ, with natural overexpression of anthocyanin-3-*O*-galactoside, and susceptible cultivar LJ43. I, phenolic acids; II, gallic acid and its derivatives; III, flavan-3-ol monomers; IV, flavan-3-ol polymers; V, flavonol glycosides; VI, anthocyanins. **b** Comparison of lesions and pink ring symptoms at different leaf positions after inoculation in cultivars ZJ and LJ43. Scale bar = 2 mm.

The resistant cultivar ‘Zijuan’ (ZJ), rich in anthocyanin-3*-O-*galactosides, a red cultivar artificially selected from *C. sinensis* var. *assamica*, was presumed to be a cultivar highly resistant to abiotic stress [16, 32, 33]. The difference in phenolic metabolites between the resistant cultivar ZJ and the sensitive cultivar LJ43 were studied using the UPLC–QqQ–MS/MS technology mentioned above (Fig. 4a) (Supplementary Data Table S1). In comparison, the total amounts of anthocyanins, phenolic acids, and PAs in the leaves of the resistant cultivar ZJ were 11.47, 2.65, and 2.17 times those in the leaves of the sensitive cultivar LJ43, respectively, while the total amounts of hydrolyzed tannins, catechins, and flavonol glycosides were only 0.74, 0.79, and 0.55 times those in the sensitive cultivar LJ43. Among them, cyanidin-3*-O-*galactoside and delphinidin-3*-O-*galactoside compounds in the resistant cultivar ZJ were 393.6 and 116.7 times those in the sensitive cultivar LJ43, respectively. The freehand sections of leaves of the resistant cultivar ZJ showed the accumulation of anthocyanins in vacuoles, which is consistent with the location of anthocyanin accumulation in infected leaves of the resistant cultivar ZC108 (Supplementary Data Fig. S1b). In contrast, the total amount of hydrolyzed tannins, PAs, and flavonol glycosides in resistant cultivar ZC108 were 1.8, 1.9, and 1.4 times those in the sensitive resistant cultivar LJ43, respectively.

A *C. camelliae* infection test was conducted on the different developmental leaves (first to fourth leaves) of the cultivars ZJ and LJ43. After infection, the lesions around the inoculation points of the resistant cultivar ZJ were smaller than those of LJ43, and the diameters of the pink rings were significantly larger than those of LJ43 (Fig. 4b). The above experiments indicate that, although rich in anthocyanins, there is also a phenomenon of anthrax inducing anthocyanin synthesis in the resistant cultivar ZJ to increase resistance. UPLC–QqQ–MS/MS was used to quantitatively detect the content of cyanidin-3-*O*-galactosides in different parts of ZJ and ZC108 after infection with *C. camelliae*. The results showed that the cyanidin-3-*O*-galactoside content in the pink ring was close to that in cultivar ZJ. This indicates that most of the pink of the pink ring comes from cyanidin-3-*O*-galactoside (Supplementary Data Fig. S3).

### Phenolic metabolic profile and gene expression profile of infected leaves

To find out the key factors that control the appearance of the pink ring, we utilized UPLC–QqQ–MS/MS technology for targeted metabolome analysis and transcriptome sequencing technology for gene expression analysis to determine the expression levels of related genes in the leaves of the resistant cultivar ZC108 from 0 to 6 dpi after infection with *C. camelliae*. The trends related to changes in phenolic content and transcript abundance of related genes in control and infected leaves inoculated with *C. camelliae* are listed in Supplementary Data Table S2 (metabolic changes) and Supplementary Data Table S3 (gene expression).

**Figure 5:**
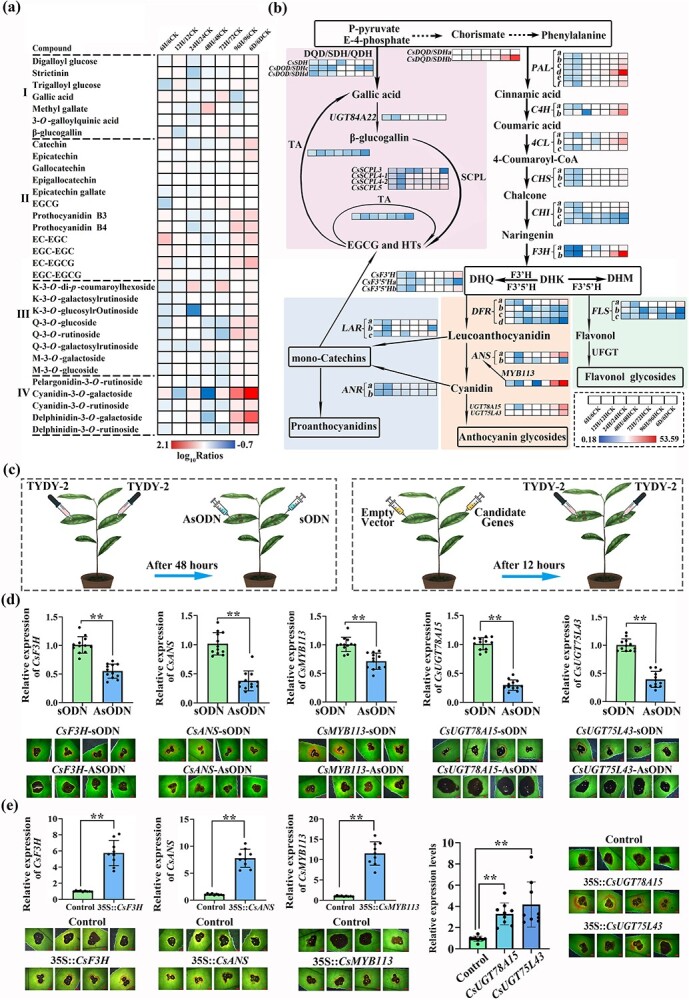
Effect of key genes involved in phenolic compound biosynthesis on the formation of pink ring symptoms. **a** Heat map analysis of the change in phenolic compound content in the infected leaves within 6 dpi. Values in the heat map are ratios of log_10_-transformed peak area values of the phenolic compounds in infected leaves to values in the control leaves. I, gallic acid and its derivatives; II, flavan-3-ol monomers and flavan-3-ol polymers; III, flavonol glycosides; IV, anthocyanins. **b** Phenolic metabolic pathway and heat map analysis of key gene expression in infected leaves within 6 dpi. Values in the heat map are ratios of FPKM values of infected leaves to those of control leaves. The galloyl derivative biosynthesis pathway, the proanthocyanidin synthesis pathway, the anthocyanin synthesis pathway, and the flavonol synthesis pathway are highlighted in pink, blue, orange, and green, respectively. **c** Schematic diagram of transient genetic overexpression and AsODN gene silencing on pink ring symptom formation. TYDY-2 is the *C. camelliae* strain used in this study for pathogenic fungus inoculation. **d** Effects of gene silencing using AsODN technology on pink ring symptoms of cultivar ZC108 at 6 dpi. Error bars represent ± standard deviation (*n* = 9). Scale bar = 2 mm. *P < 0.05, **P < 0.01. **e** Effects of gene overexpression using *Agrobacterium*-mediated transient transformation on pink ring symptoms on leaves of susceptible cultivar LJ43 at 4 dpi. Scale bar = 2 mm; *P < 0.05, **P < 0.01.

We observed that the impact of mechanical injury on the expression of related genes in leaves was primarily observed within 12 h after inoculation, followed by a sharp decline in the expression of most genes. Due to the combined effect of *C. camelliae* infection and mechanical damage, most genes in infected leaves showed a pattern of initial high expression followed by a decrease, and then a subsequent increase within the 0- to 6-dpi period (Supplementary Data Table S3). Therefore, we focused on the metabolic and gene expression changes between 3 and 6 dpi. The heat map in Fig. 5a illustrates the ratio of phenolic substance content (peak area value) in infected leaves to uninfected leaves within 0–6 dpi (Supplementary Data Table S2), while Fig. 5b displays the corresponding transcript abundance of key genes (FPKM value).

The heat map illustrates that the increase in polyphenol content in leaves inoculated with *C. camelliae* occurred at 3–4 dpi and reached the highest level at 6 dpi (Fig. 5a). The contents of anthocyanins, flavonols, and PAs in leaves showed increasing trends within 3–6 days after inoculation. Notably, the contents of cyanidin-3-*O*-galactoside and delphinidin-3-*O*-galactoside increased by 29.89 and 13.68 times, respectively, from 3 to 6 dpi. The content of PAs also doubled during this period. Interestingly, the primary phenolic substances in tea plants, such as flavan-3-ol gallates like EGCG and catechin EGC with a B-ring trihydroxy group, exhibited minimal increases. The above data indicated that the biosynthesis of anthocyanins, PAs, and flavone glycoside compounds may be upregulated by *C. camelliae* infection.

In the pathways of phenylpropane, flavonoid, and galloyl derivative biosynthesis in tea plants, the structural genes are composed of gene families, each family containing at least two paralogous genes [7]. Based on transcriptome sequencing data, the expression of at least one paralogous gene in most steps of these pathways exhibited upregulation during infection, except for the galloyl derivative biosynthesis pathway (Supplementary Data Table S3, Fig. 5b). For example, the expression levels of *CsDQD/SDHb*, *CsPALd*, *CsC4Hb*, *CsC4Hc*, and *F3Hb* in infected leaves at 6 dpi were 42.1, 53.6, 12.4, 28.2, and 42.3 times higher than those in the control. This indicated that the expression of these genes was strongly induced by *C. camelliae*.

*CsFLS*s, *CsANS*s, *CsLAR*s, and *CsANR*s catalyze the key step of flavonol, anthocyanin, and catechin biosynthesis [34–36]. The expression levels of *CsDFRa*, *CsF3'H*, *CsLARc*, *CsANSa*, and *CsFLSa* in infected leaves at 6 dpi were 7.8, 10.2, 6.0, 24.3, and 7.0 times higher than those of the control (Supplementary Data Table S3, Fig. 5b). Among them, the expression level of the *CsANSa*, which catalyzes the synthesis of anthocyanins, was the highest. Gene changes in the flavonoid upstream pathway were more dramatic than in the flavonoid downstream pathway.

*CsDQD/SDHc*, *CsDQD/SDHd*, *CsUGT84A22*, *CsSCPL4*, *CsSCPL5*, and *CsTA*s are the catalytic enzymes in the galloyl derivative biosynthesis pathway [11, 12]. The expression of these genes was not induced by infection with the *C. camelliae* (Fig. 5b, Supplementary Data TableS3). The expression level of *CsTA*, which has been proven to promote the synthesis of gallic acid derivatives, was lower than that of the control (Fig. 5b).

Transcription proteins such as MYB, BHLH, and WD40 are known to play a role in regulating the biosynthesis of flavonoids. Previous studies have demonstrated that MYB members belonging to the sixth subgroup serve as positive regulatory transcription factors in anthocyanin synthesis [15, 16]. Four MYB transcription factors (Supplementary Data Table S3) belonging to the sixth subgroup were found in the transcriptome database, and only the expression of the *CsMYB113* gene was upregulated by *C. camelliae* infection (Supplementary Data Fig. S5a and b). The expression level of *CsMYB113* in infected leaves at 6 dpi was 12.5 times higher than that of the control. Furthermore, the experiment further confirmed that *CsMYB113* stimulated the expression of the CsANSa promoter (Supplementary Data Fig. S5c).

UDP-glycosyltransferases (UGTs) are known to catalyze the glycosylation of anthocyanins. Figure 2 shows that the content of cyanidin-3*-O-*galactosides increased significantly in leaves infected by *C. camelliae*. Hence, the primary objective of our research was to identify the specific UGTs involved in the galactosylation of anthocyanins. The gene expression profile showed that the expression of 24 CsUGTs was upregulated by *C. camelliae* (Supplementary Data Fig. S4a). They were classified into 11 UGT subgroups (Supplementary Data Fig. S4b). We expressed the recombinant proteins of *CSS0010045* (*CsUGT78A15*), *CSS0043878* (*CsUGT75L43*), *CSS0020241*, *CSS0020068*, *CSS0000054*, *CSS0019985*, and *CSS0046076* using the prokaryotic expression system, and carried out the enzyme test *in vitro*. The results showed that only the recombinant CsUGT78A15 and CsUGT75L43 proteins could catalyze the galactosylation of anthocyanidins, cyanidin and delphinidin (Supplementary Data Fig. S4c). The expression levels of *CsUGT78A15* and *CsUGT75L43* in the infected leaves at 6 dpi were 24.3 and 16.9 times higher than control.

### Identification of key resistance genes via genetic manipulation approaches

Whether the genes mentioned above regulate the formation of the pink ring and participate in the resistance of tea plants to anthracnose requires direct evidence for linking genetic manipulation to the resistance. We used the antisense oligonucleotide (AsODN)-mediated gene expression inhibition test and our established tea plant gene transient expression system to verify the function of key genes in the formation of the pink ring [12]. The AsODN-mediated gene expression inhibition test was carried out in the resistant cultivar ZC108, and the transient genetic expression test was performed as in Fig. 5c. Primer sequences used in the test are listed in Supplementary Data Table S4. After the AsODN leaves were inoculated with *C. camelliae*, the lesion expanded, and the pink ring appeared later or even disappeared compared with sODN (sense oligonucleotide) leaves (Fig. 5d). In particular, after *CsUGT78A15* and *CsUGT75L43* in the leaves were downregulated by interference, the lesions on the leaves expanded significantly. The results of qRT–PCR showed that the gene expression levels of *CsF3Ha*, *CsANSa*, *CsUGT78A15*, *CsUGT75L43*, and *CsMYB113* in tea leaves treated with their specific AsODNs were downregulated by 29–71%.

Next, under the action of the 35S promoter, *CsF3Ha*, *CsANSa*, *CsUGT78A15*, *CsUGT75L43*, and *CsMYB113* were overexpressed in tea leaves using a transient expression system (Fig. 5e). Quantitative results of qRT–PCR showed that the expression of these genes was significantly upregulated. Compared with the control, overexpressing the above genes led to earlier formation of a pink ring on the leaves, and the area of the lesion was reduced.

The above genetic manipulation results showed that the expression levels of *CsF3Ha*, *CsANSa*, *CsUGT78A15*, *CsUGT75L43*, and *CsMYB113* genes were indeed positively correlated with disease resistance and were also related to the formation of the pink ring induced by *C. camelliae*. In addition, *CsMYB113* directly activated the transcription of GhMYB25 for early fiber development. *CsMYB113* directly activated the transcription of *CsANSa* and promoted the accumulation of anthocyanin-3*-O-*galactosides around the invasion sites of *C. camelliae* (Supplementary Data Fig. S5).

### Screening of key signal pathways regulating the appearance of the pink ring

The results of transcriptome sequencing showed that the expression of key genes in the JA, salicylic acid (SA), and ethylene (ET) biosynthetic and signaling pathways, such as key genes for lipoxygenases (LOXs), 12-oxophytodienoate reductases (OPRs), MYC2 transcription factors, ACC oxidase (ACO), ethylene-responsive factors (ERFs), phenylpropane lyase (PAL), and PBS3 (a GH3 acyl adenylase family enzyme), were significantly upregulated during infection of tea leaves by *C. camelliae* (Supplementary Data Table S5, Supplementary Data Fig. S6). To verify whether JA, SA, and ET hormones participate in the induction of the formation of the pink ring, we tried to test the effects of different concentrations of exogenous JA, SA, and ET hormones on the formation of the pink ring. The results showed that these exogenous hormones did not successfully induce the production of the pink ring but tended to increase expansion of the lesion (Supplementary Data Fig. S7).

**Figure 6:**
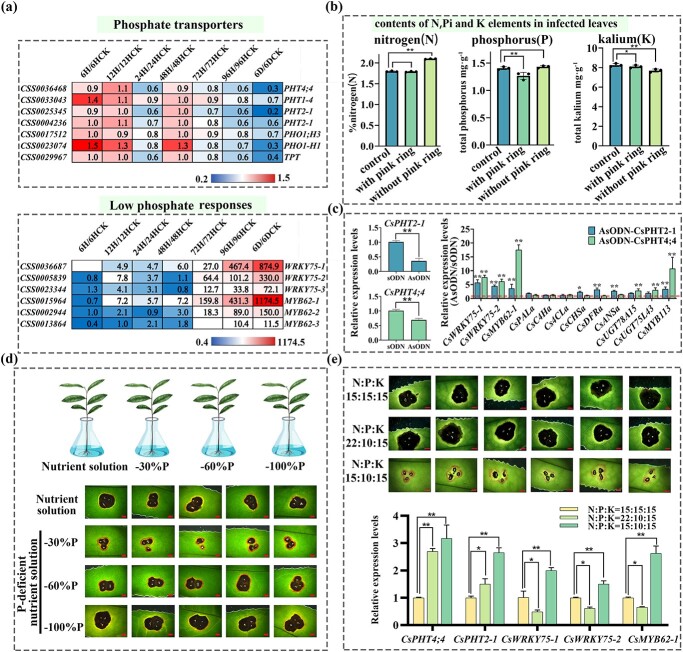
Pi deficiency induced by anthracnose involved in pink ring symptom formation. **a** Expression pattern of genes involved in phosphorus transport and encoding low Pi response transcription factors in infected leaves within 6 dpi. FPKM values of key genes for the experimental/control ratio are represented by a heat map. **b** Total N/P/K content in infected leaves with and without symptoms of pink ring. Error bars represent ± standard deviation (*n* = 3). *P < 0.05, **P < 0.01. **c** Effects of silencing of *CsPHT*s using AsODN technology on related gene expression in leaves of tea plants. Error bars represent ± standard deviation (*n* = 3). *P < 0.05, **P < 0.01. **d** Effect of Pi deficiency treatment on pink ring symptoms of healthy tea branches. Scale bar = 2 mm. **e** Effects of three slow-release base fertilizers on pink ring symptoms and related gene expression levels on healthy 2-year old tea seedlings. Error bars represent ± standard deviation (*n* = 6). Scale bar = 2 mm. *P < 0.05, **P < 0.01.

The expression level changes in some genes in response to phosphate (Pi) deficiency in the transcriptome have attracted our attention. For example, the expression levels of *CsMYB62*s and *CsWRKY75*s were significantly upregulated, and some phosphate transporters, including the PHOSPHATE TRANSPORTERS (PHTs) family and the PHOSPHATE1 (PHO1) family, were downregulated in infected leaves (Supplementary Data Table S6, Fig. 6a). According to reports, the induction of *MYB62* is a specific response in the leaves during Pi deprivation [37], or regulated anthocyanin biosynthesis in *Malus* under low-nitrogen conditions [38]. The expression of transcription factor *WRKY75* members in some plants was induced by Pi deficiency and participated in the positive regulation of anthocyanins [39, 40].The phosphate transporters, including the PHT1–5 family and PHO1 family, the SYG1/Pho81/XPR1 (SPX) domain-containing protein family, and the SULTR-like family members, are likely involved in Pi uptake, transport, and mobilization [41]. This data suggests that the appearance of the pink ring may be related to the Pi deficiency induced by *C. camelliae* in infected leaves.

To confirm the above speculation, the total N, Pi, K contents of infected leaves with and without the pink ring were determined. The results confirmed that the total phosphate content of infected leaves with the pink ring was lower than that of infected leaves without the pink ring, while the trend of changes in N element content was opposite (Fig. 6b).

We studied the effects of N and K deficiency on the formation of the pink ring and disease resistance after infection with *C. camelliae*. The effects of N and K deficiencies on pink ring symptoms were investigated in hydroponic experiments. Healthy tea seedlings were cultured in complete nutrient solution and N- or K-deficient nutrient solution and then inoculated with *C. camelliae*. It was found that N and K deficiencies did affect the disease resistance of tea plants, and this resulted in the enlargement of lesions and the appearance of the pink ring. In addition, tea tree branches were treated hydroponically with 90 μM sucrose for 48 h and then inoculated with *C. camelliae*. It was found that the pink ring appeared earlier and the area of the lesions was smaller after sucrose treatment (Supplementary Data Fig. S8).

In order to determine that phosphorus transporters did indeed affect the expression of downstream genes *WRKY75s* and *MYB62s*, as well as anthocyanin-related genes, transient interference tests were performed on the expression of two *CsPHT* genes. When the expressions of *CsPHT*s in tea leaves were momentarily disrupted, the expression of the above *CsMYB62-1*, *CsWRKY75*s, and *CsMYB113* were significantly upregulated, indicating that their gene expression responded to Pi deficiency (Fig. 6c).

Multiple repeated tests have shown that the total phosphate content of leaves with pink ring was ~10% lower than that of normal leaves. Further test results showed that with culture medium with 30% Pi reduction there was indeed a reduction in disease spots and a more pronounced pink ring symptom on infected leaves, compared with the other three treated branches, including those in fully nutrient solution, 60% Pi reduction, and 100% Pi reduction (Fig. 6d). Further experiments were conducted by applying three slow-release base fertilizers to the soil of 2-year-old tea seedlings. Six months later, anthracnose was inoculated on the leaves of tea seedlings and the results were observed. The lesions area of the cuttings grown under the culture condition of N: P: K = 15:10:15 after being infected with anthracnose are the smallest compared to the cuttings grown under the culture conditions of N: P: K = 15:15:15 and N: P: K = 22:10:15., and the expression levels of *CsPHT*s, *CsWRKY*s, and *CsMYB62-1* were significantly higher than those of the other two treatments (Fig. 6e). The above experiments showed that there is indeed a phenomenon of anthrax-induced phosphate deficiency, which may be one of the reasons for the appearance of pink ring symptoms.

**Figure 7:**
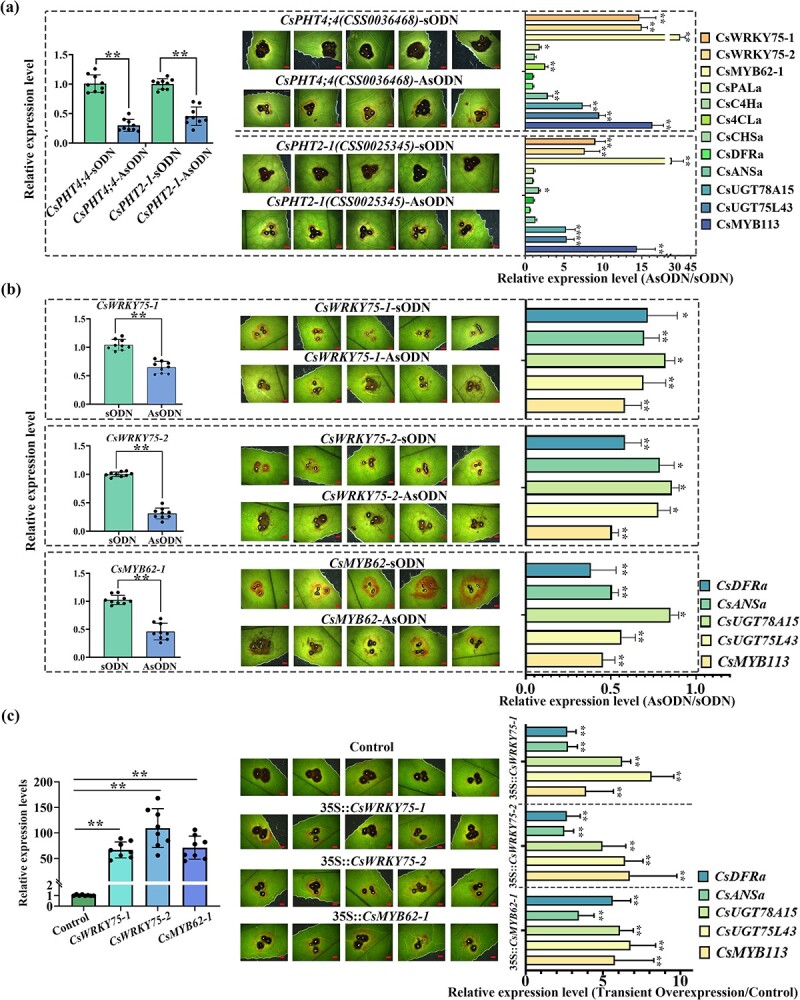
Functional verification of genes involved in phosphorus transport and encoding low Pi response transcription factors on the formation of pink ring symptoms. **a**, **b** Functional verification of genes involved in phosphorus transport and encoding low Pi response transcription factors using AsODN technology on pink ring symptoms and related gene expression at 6 dpi. Error bars represent ± standard deviation (*n* = 6). Scale bar = 2 mm. *P < 0.05, **P < 0.01. **c** Functional verification of genes involved in low Pi response transcription factor gene overexpression using *Agrobacterium*-mediated transient transformation on pink ring symptoms and related gene expression at 6 dpi. Error bars represent ± standard deviation (*n* = 6). Scale bar = 2 mm. *P < 0.05, **P < 0.01.

AsODN-mediated *CsPHT4;4*, *CsPHT2-1*, *CsWRKY75-1*, *CsWRKY75,2*, and *CsMYB62-1* gene expression inhibition tests and subsequent inoculation with *C. camelliae* are shown in Fig. 7a and b. When the expression of *CsPHT4;4* and *CsPHT2-1* genes was downregulated in infected leaves, the expression levels of these genes, including *CsWRKY75-1*, *CsWRKY75-2*, *CsMYB62*, *CsMYB113*, *CsUGT78A15*, and *CsUGT75L43*, were significantly upregulated, indicating that their expression was indeed induced by Pi deficiency. When the expression of *CsWRKY75-1*, *CsWRKY75-2*, and *CsMYB62* was downregulated in infected leaves, the expression of *CsMYB113*, *CsUGT78A15*, *CsUGT75L43*, *CsANSa*, and *CsDFRa* was upregulated to varying degrees. On the contrary, the overexpression of *CsWRKY75-1*, *CsWRKY75-2*, and *CsMYB62* upregulated the expression levels of the aforementioned genes in the susceptible cultivar LJ43 (Fig. 7c). The above results indicated that *CsWRKY75s* and *CsMYB62* were involved in the anthocyanin synthesis induced by anthrax in some way.

**Figure 8:**
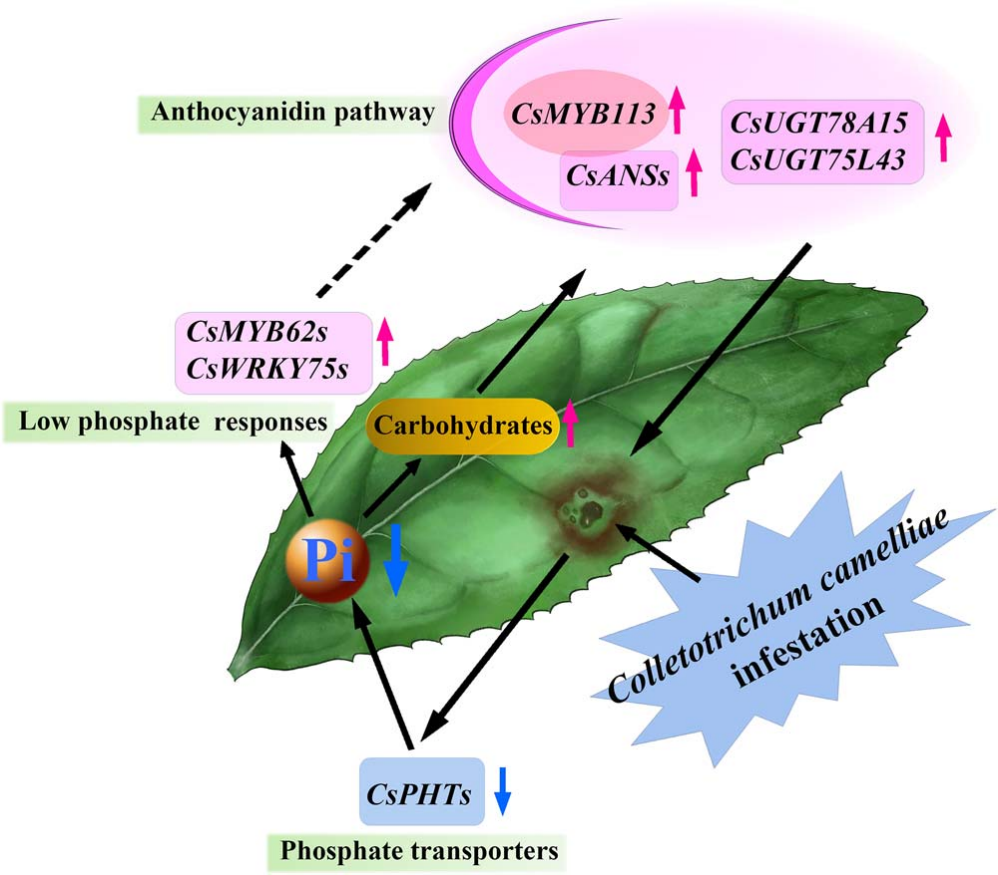
Model of phosphate deficiency induced by infection promotes synthesis of anthracnose-resistant anthocyanin-3-*O*-galactoside phytoalexins.

Based on the research results in this article, a pattern diagram was drawn (Fig. 8): when the resistant variety of tea plants is infected with anthrax, the gene expression of Pi transporters such as *CsPHT*s decreases, and the expression of Pi-deficiency response genes *CsWRKY75* and *CsMYB62* increases in the infected leaves. This leads to the upregulation of gene expression in the anthocyanin synthesis pathway of cells around the infection site, which accumulates anthocyanins, thereby hindering the diffusion of mycelium. The qRT–PCR results showed that the expression of the phosphate transporter protein CsPHTs in leaves around the invasion site of the pathogen was 5–10% of that in uninfected areas. The expression levels of Pi-deficient response genes *CsWRKY75*s and *CsMYB62-1* were 250–6000 times higher in leaves around the invasion site than in uninfected leaves. It is suggested that it is the localized physiological phosphate deficiency caused by pathogen invasion that led to anthocyanin-3*-O-*galactoside accumulation (Supplementary Data Fig. S9).

## Discussion

### Anthocyanin synthesis induced by pathogenic microorganisms

Due to the convenience of observation attributable to its red color, anthocyanin biosynthesis and its regulation mechanism still attract a lot of attention, and have become the major target of genetic manipulation and genome editing [42–44]. Anthocyanin accumulation in different plants has different tissue-specific metabolite profiling. Anthocyanins can be detected in the petals, leaves, seed coats, and fruits of different plants [45, 46]. Anthocyanin synthesis is induced by some abiotic stress processes, including UV irradiation, low temperature, drought, salinity, and nutrient deficiency [47–50]. Anthocyanins, like most phenolic compounds, have phenolic hydroxyl groups. Therefore, from the perspective of their mechanism of action, anthocyanins usually have the function of scavenging free radicals and reactive oxygen species, thus enhancing the resistance of plants to abiotic stresses.

In comparison, several studies have reported that the relationship between anthocyanin accumulation and biotic stress is less than that between anthocyanin accumulation and abiotic stress. It is well known that anthracnose induces the accumulation of anthocyanins in maize [51], and bacteria induce anthocyanin accumulation in cotton [31].

In most cases it is difficult to determine whether the colorless phytoalexins induced by microorganisms are formed at the infection site or in the normal cells around the infection site. However, there is no method to detect the subcellular localization of phytoalexins. Recently developed MS imaging technology is expected to become a powerful tool to explore the subcellular localization of phytoalexins [52]. Due to the visible characteristics of anthocyanins, it is convenient to assess the subcellular localization of phytoalexins such as anthocyanins. It was found that in resistant sorghum varieties, when infected with pathogenic *Colletotrichum* fungi, red phytoalexin was synthesized in the first epidermal cells infected by fungi and formed subcellular inclusions in them rather than in cells around the infection site [31].

In this study, we found that the location of anthocyanin accumulation after induction by *C. camelliae* in tea plants is different from that in sorghum. It is difficult to observe a phenomenon similar to that of sorghum in tea, because sorghum is induced by the fungus to accumulate anthocyanins in its epidermal cells. The pink ring induced by *C. camelliae* was located around the infection point, with a green isolation zone in the middle (Figs 1 and2); the microanatomy showed that the part containing anthocyanins was the mesophyll tissue rather than the epidermal tissue (Fig. 2, Supplementary Data Fig. S1). The time when the pink ring can be observed by the naked eye is 3–5 days after infection; metabolic detection data also showed that a significant increase in anthocyanin content occurred in leaves 4 days after infection (Fig. 3).

This phenomenon clearly shows that the induced synthesis of anthocyanin phytoalexins in tea plants is located in the mesophyll cells around the infection point. The positions of phytoalexins produced in different plants are indeed very different. Anthocyanin synthesis induced by bacterial infection is found around epidermal cells as a stomatal complex [51]. These positional differences may indicate differences in their disease resistance mechanisms. However, from the perspective of disease resistance mechanisms, the small-scale and high-concentration accumulation of phytoalexin compounds, such as anthocyanins, is conducive to inhibiting the spread of pathogens. The phenomena we observed in this study prove the same point (Fig. 3c–e).

We are also concerned with a report about the favorable effect of caffeine in inhibiting *Colletotrichum* [53]. Metabolite assay data showed that caffeine content in tea plants did not increase with fungal infestation. The peak area of theobromine was even reduced (Supplementary Data Fig. S10). This suggests that the biosynthesis of caffeine and theobromine in tea plants did not undergo an induced increase as a result of *C. camelliae* infestation. Despite *in vitro* tests demonstrating that the antimicrobial activity of caffeine, based on its characteristics, is not induced by *C. camelliae*, we concluded that caffeine in tea plants is not the ‘phytoanticipins’ or ‘phytoalexin’. On the other hand, cyanidin-3-*O*-galactoside biosynthesis in tea plants was significantly induced by *C. camelliae*. Therefore, cyanidin-3-*O*-galactoside rather than caffeine is the ‘phytoalexin’ induced by fungi in tea plants.

### Key genes of anthocyanin synthesis induced by pathogenic microorganisms

Although phytoalexin is induced by biotic and abiotic stresses, the mechanism involved remains unclear. It is still confusing what happens among plants, phytoalexins, and pathogens [54]. For the environmental conditions in which the pathogenic fungus *Colletotrichum* grows, water and temperature are two important factors; therefore, in warm and humid areas anthracnose is a serious disease in plants.

3-Deoxyanthocyanidin is a phytoalexin from sorghum and is specifically localized around fungal infections [54]. Plant antitoxins accumulate more rapidly in infected cells of disease-resistant varieties than in susceptible varieties, preventing the proliferation of fungal hyphae throughout the tissues [55]. It is reported that LJ43 is sensitive to anthracnose, while ZC108 is resistant to it [30]. The test results in this study also show that varieties with strong resistance, such as ZC108 and ZJ, are more likely to have pink circle symptoms than the susceptible cultivar LJ43 (Fig. 2a). However, we also found that the healthy seedlings of the susceptible cultivar LJ43 easily produced a pink ring, indicating that the cultivation condition might determine the strength of plants’ resistance to diseases. There may be cross-talk in the molecular signal pathways between nutrient uptake and disease resistance [56, 57].

There are many structural and transcription factor genes involved in the flavonoid and anthocyanin synthesis pathway. More than 30 structural genes are involved in tea plants from the beginning of the phenylpropanoid pathway to the end of the flavonoid pathway, and almost every step is controlled by a gene family. The identification of key genes involved in anthocyanin synthesis is the goal of researchers and the object of gene editing [42]. In particular, the identification of key genes related to plant resistance has a good application prospect. In this paper, the genetic manipulation approaches combined with the phenomenon of visible ring strengthening or weakening provide an easy way to identify resistance genes (Fig. 5). The change in gene expression changed the formation of the pink ring and the size of the lesion; this included the upstream gene *F3H* of the flavonoid pathway, the terminal gene *CsANS* of anthocyanin synthesis, the genes *CsUGT78A15* and *CsUGT75L43* involved in anthocyanin galactosylation, and the expression of the transcription factor gene *CsMYB113* involved in anthocyanin synthesis. Notably, when the expression of *CsUGT78A15* and *CsUGT75L43* genes related to anthocyanin glycosylation changed, the lesion enlarged.

### Signal pathway involved in anthocyanin synthesis induced by pathogenic fungus *C. camelliae* in tea plants

The signal pathway involved in the anthocyanin synthesis induced by *C. camelliae* in tea plants could be a future research direction. Transcriptome data showed that the genes of the JA, SA, and ET signal pathways and other pathways were activated to varying degrees when tea leaves were infected with *C. camelliae* (Supplementary Data Fig. S7). However, exogenous JA, SA, and ET treatment did not successfully induce the appearance of the pink ring or reduce lesion size (Supplementary Data Fig. S7). Nevertheless, this does not mean that these pathways do not involve the resistance pathway of plants, but they do not necessarily participate in the formation of the pink ring.

We speculate that one of the main factors for inducing the pink ring in infected leaves of tea plants is the physiological Pi deficiency caused by infection with *C. camelliae*, namely, pathogenic fungus *C. camelliae*-induced Pi deficiency (Fig. 6). This conjecture is based on the following facts. First, some genes responding to Pi deficiency were significantly upregulated in infected leaves with the pink ring, such as *CsMYB62*s and *CsWRKY75*s (Fig. 6a). An experiment showed that *AtMYB62* gene expression was specifically upregulated in the leaves during Pi starvation [37]. This suggests that the appearance of a pink ring may be related to the decrease in Pi content in the infected leaves. Next, the quantitative detection results confirmed that the total Pi content of infected leaves with the pink ring was lower than that of infected leaves without the pink ring, although these leaves were from the same cultivar (Fig. 6b). Third, when the expression of *CsPHT*s in tea leaves was momentarily disrupted, the expressions of the above *CsMYB62*s and *CsWRKY75*s were indeed significantly upregulated, indicating that their gene expression responded to Pi deficiency (Fig. 6c). Compared with the infected leaves in complete culture medium (containing 100% Pi) or under the N:P:K = 15:10:15 slow-release fertilizer condition, the symptoms of pink ring on the infected leaves under these Pi-deficiency mediums were more pronounced (Fig. 6d and e).

Phosphorus homeostasis in plants is affected by Pi absorption, transport, and metabolism [58]. Transcription factors, including *PHR1*, *PHL1*, *WRKY75*, and *MYB62*, are reported to be low Pi response factors and play critical roles in the control of Pi starvation responses [59, 60]. We speculate that the *PHT* gene family is one of the key factors in regulating Pi homeostasis in infected leaves. The transcriptome sequencing data showed that 33 genes annotated with phosphate transporters *CsPHT* and *CsPHO* could be detected in tea plants, and the transcript levels of 21 genes were almost undetectable in leaves (Supplementary Data Table S6). The transcription levels of 8 of the remaining 12 genes were significantly lower in leaves infected with *C. camelliae* than in those of the control, including *CsPHT1-4*, *CsPHT2-1*, *CsPHT4;4*, and *CsPHO1;H3* (Fig. 6a). The subcellular localization, expression patterns, and regulation of P homeostasis balance in plants, and tolerance to stress of different phosphate transporter gene families have been partially studied [61]. When these *CsPHT*s were downregulated in leaves infected with *C. camelliae*, the expression levels of downstream transcription factor genes regulated by them were upregulated and corresponding changes in the pink ring symptom occurred (Fig. 7a).

All the above results indicate that the phenomenon of *Colletotrichum*-induced Pi deficiency occurs in the process of tea plants being infected with this pathogen (Fig. 8). Plants overexpressing *OsPT8* (a high-affinity transporter for both Pi and arsenate) were more susceptible to fungal and bacterial rice pathogens [57]. Excessive Pi fertilization increases the sensitivity of rice to fungal diseases [62]. Research on Pi deficiency in tea plants has also been reported, and the transcription factors *PHR1*, *PHO1*, and *SPX2* were upregulated under Pi-deficient conditions in each light treatment group [63]. This suggests that these transcription factors may play an important role in the response of tea plants to different stresses. There are still many questions to be solved, such as whether the pink ring phenomenon is universal and whether other fungi or bacteria can also induce tea leaves to show a pink ring.

## Materials and methods

### Plant materials and culture conditions

The 2-year-old tea cultivars selected for the inoculation test with *C. camelliae* included *Camellia sinensis* var. *sinensis* (CSS) cultivars ‘Qiancha8’ (Q8), ‘Yibin6’ (Y6), ‘Longjing43’ (LJ43), ‘Zhongcha108’ (ZC108), and ‘Huangjinjia’ (HJJ), and *C. sinensis* var. *assamica* (CSA) cultivar ‘Zijuan’ (ZJ). These cultivars were cultivated in the greenhouse belonging to the experimental garden of the Anhui Agricultural University in Hefei, Anhui, China (31°30′ N, 177°17′ E). The phytotron was set to a 14/10 h light/dark photoperiod, 25/20°C light/dark temperature, and 85% relative humidity. Healthy plants were selected for the inoculation test.

### Anthracnose inoculation

The pathogenic isolate (TYDY-2) used in the study was identified as belonging to *C. camelliae*, which is highly virulent to tea plants [22]. The *C. camelliae* strain was first cultured on potato dextrose agar (PDA) medium for 5 days at 28°C, and the spores were then collected by centrifugation for 10 min at 6000 × g min^−1^. The collected spores were resuspended in sterile water and adjusted to a concentration of 10^6^ spores ml^−1^ for subsequent inoculation tests.

Fifty microliters of spore suspension was inoculated into tea leaves after trauma with a sterile needle. The control plants were inoculated with the same amount of sterile distilled water. The inoculated leaves were coated with a film to maintain high humidity and facilitate the growth of fungi. The film was removed after 24 h and this was followed by normal culture.

### Observation of surfaces and paraffin sections of infected leaves

The symptoms of the lesion in leaves were recorded using a stereo microscope (Zeiss, Germany). The microscopic structures of the hand and paraffin sections of leaves infected with *C. camelliae* were observed using an optical microscope (Nikon Eclipse, Japan). The protoplasts were extracted from the pink ring tissues according to the procedure mentioned in a previous study and recorded using an optical microscope (Nikon Eclipse, Japan) [64].

The fixed diseased leaves were placed into the dehydrator and dehydrated with an alcohol gradient . Wax-soaked tissue was embedded in an embedding machine (Wuhan Junjie Electronics Co., Ltd, Wuhan, China). After cooling, the modified tissue chip wax block was sliced on a paraffin slicer (Shanghai Leica Instrument Co., Ltd, Shanghai, China) at a thickness of 4 μm. The sections were dewaxed with absolute ethanol and then stained with Safranin O solution and plant solid green staining solution, respectively The samples were decolorized with a series of graded ethanol concentrations after each staining. Finally, the tissue sections were mounted with neutral balsam and observed under a microscope (Nikon Eclipse, Japan), and images were taken using the camera attached to the microscope.

### Extraction and detection of phenolic substances in tea leaves

Samples from three replicates of leaves and control were collected at 12 hours post-inoculation (hpi) and 1, 2, 3, 4, and 6 dpi. The total polyphenols in tea samples were identified according to a published method [6]. First, 0.06 g of freeze-dried tea sample was extracted using ultrasonication with 4 ml of 80% methanol solution. The extracts were then centrifuged at 12 000 rpm for 10 min to collect the supernatant. The residues were extracted three times. The UPLC–MRM–MS/MS technique was used for qualitative and quantitative detection of phenolic compounds. The collected supernatants were diluted to 5 mg·ml^−1^ and filtered through a 0.22-μm filter membrane prior to UPLC–MRM–MS/MS analysis.

The elution solution and procedure involved in the LC technology, as well as the fragmentation voltage and collision energy involved in the MS/MS technology during UPLC–MRM–MS/MS analysis, and the qualitative and quantitative methods for phenolic compound estimation were implemented using our published procedures [6]. MS and MS/MS data files were obtained using Qualitative Analysis 10.0 analysis.

### *Agrobacterium*-mediated transient gene expression test in tea leaves

Transient genetic transformation of the target genes into tea leaves was carried out according to a method published previously [12]. Full-length cDNAs of the target genes were cloned into the expression vector pCAMBIA1305 containing the GFP tag to construct the donor plasmids. The primers for candidate gene cDNA clones are shown in Supplementary Data Table S8. The donor plasmids were introduced into *Agrobacterium tumefaciens* strain GV3101 (pSoup19). *Agrobacterium* cells transformed with target gene constructs were grown in liquid LB medium and then resuspended in injection medium. In addition, the injection medium contained 100 μM acetylsyringone, 10 mM MES, and 10 mM MgCl_2_.

Leaves transiently overexpressing the target gene after 12 h were inoculated with *C. camelliae*, as described above in the section Anthracnose inoculation. The symptoms of infected leaves were observed and recorded according to the method described above in the section Observation of surfaces and paraffin sections of infected leaves. Gene expression levels were detected 48 h after injection using qRT–PCR analysis. At least three biological replicates of each treatment were carried out.

### AsODN-mediated gene expression inhibition test in tea leaves

AsODN fragments of target genes were designed using SOLIGO software [65], and the corresponding sODN fragments were selected for use in the control tests. These fragments were chemically synthesized by Tongyong Biosystems Company, and their primer sequences are shown in Supplementary Data Table S4. After 48 h of inoculation by *C. camelliae*, 1 ml of 60 μM AsODN or sODN primer was injected into the infected leaves. Gene expression levels were detected 48 h after injection using qRT–PCR analysis, and symptoms were recorded using a stereo microscope. The primers for qRT-PCR are displayed in Supplementary Data Table S7. At least five biological replicates of each treatment were carried out.

### Detection of N/P/K element content in susceptible leaves

All N/P/K measurements were carried out with a Thermo Fisher Scientific iCAP TQ ICP–MS/MS instrument (ThermoFisher, Waltham, USA). A sample of 0.1–0.5 g (with an accuracy of 0.0001 g) was weighed and placed in the Teflon vessel. Concentrated nitric acid (5 ml) was added. The temperature was increased linearly to 190°C and kept there for 20 min. The sample was placed on an acid extractor to remove acid, and reduced to nearly dry. The inner cover was rinsed with a small amount of water; the sample was adjusted to a constant volume of 10 ml with 1% nitric acid, and mixed well for later use. At the same time, a blank test was performed.

The tuning fluid was used to adjust and optimize the instrument parameters before each experiment to make the sensitivity, resolution, and stability meet the requirements. The main working parameters were as follows: tuning mode, STD/KED; dwell time, 0.1 s; peristaltic pump speed, 40 rpm; sample introduction time, 40 s; plasma power, 1550 W; sampling depth, 5.0 mm; nebulizer flow, 0.98 l·min^−1^; cool flow, 14.0 l·min^−1^; auxiliary flow, 0.8 l·min^−1^; spray chamber temperature, 2.7°C; torch horizontal position, 0.16 mm; torch vertical position, −0.53 mm; He flow, 4.55 ml·min^−1^; O_2_ flow, 0.3125 ml·min^−1^; D1 lens, −350 V; D2 lens, −350 V; repeat times, 3.

## Supplementary Material

Web_Material_uhad222
