# Supplementary material for: Phosphate deficiency induced by infection promotes synthesis of anthracnose-resistant anthocyanin-3-O-galactoside phytoalexins in the Camellia sinensis plant
Source: Hortic Res. 2023 Dec 5;10(12):uhad222. doi: 10.1093/hr/uhad222 (PMC10709544; doi:10.1093/hr/uhad222)
Supplement: Web_Material_uhad222 [file web_material_uhad222.zip › Supporting Information.docx]

## Supporting Information

Article title: **Phosphate deficiency induced by infection promotes synthesis of anthracnose-resistant anthocyanin-3*-O-*galactoside phytoalexins in *Camellia sinensis* plant**

Authors: Tongtong Li^1^, Shenrong Wang^2^, Dandan Shi^2^, Wen Fang^2^, Lixin Zhang^3^, Ting Jiang^2^, Yajun Liu^2^*, Liping Gao^2^*, and Tao Xia^1^*

The following Supporting Information is available for this article:

**Fig. S1** Cyanidin-3*-O-*galactosides accumulation patterns in “pink rings” were analyzed by slice observation and protoplast separation. (a) Schematic diagram of the sampling site of the “pink rings”. (b) White light observes the longitudinal section of the “pink rings” and the protoplast of “pink rings”. (c) White light observes the longitudinal leaf section of ZJ and the protoplasts of ZJ.

**
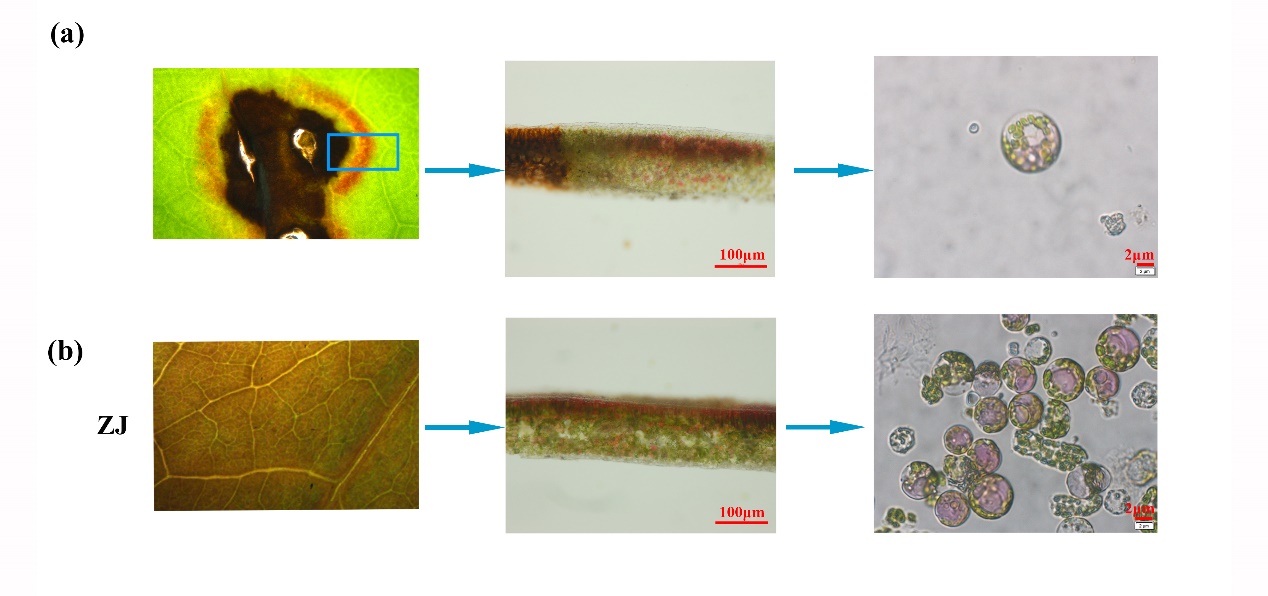
**


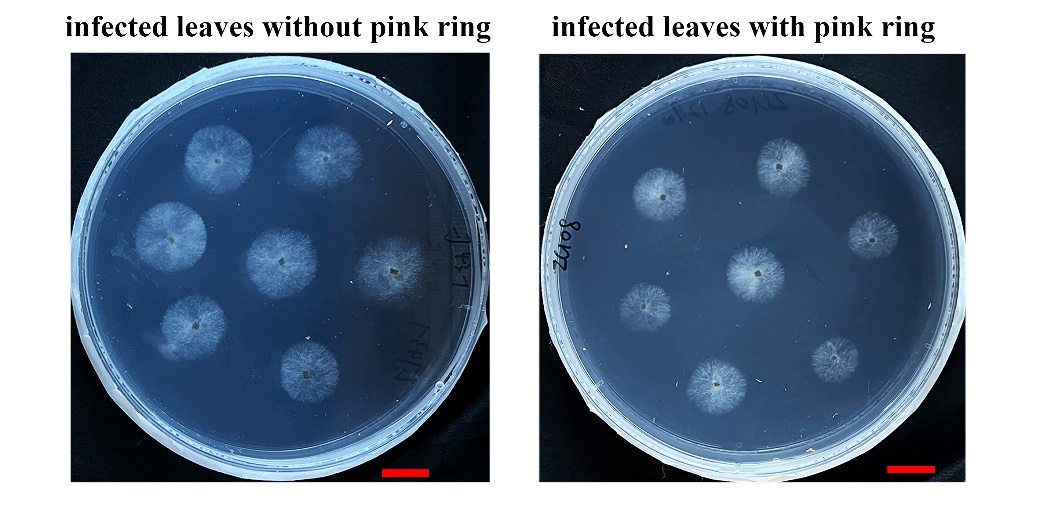
**Fig. S2** Inoculation of “infected leaves with pink ring” and “infected leaves without pink ring” diseased leaves at 6 dpi on PDA medium, incubation at 25 °C, and pictures were taken after 2 days of inoculation.

**Fig. S3** UPLC-QqQ-MS/MS was used to quantitatively detect the content of cyanidin-3*-O-*galactosides in different parts of ZJ and ZC108 after infection with *C. camelliae*.


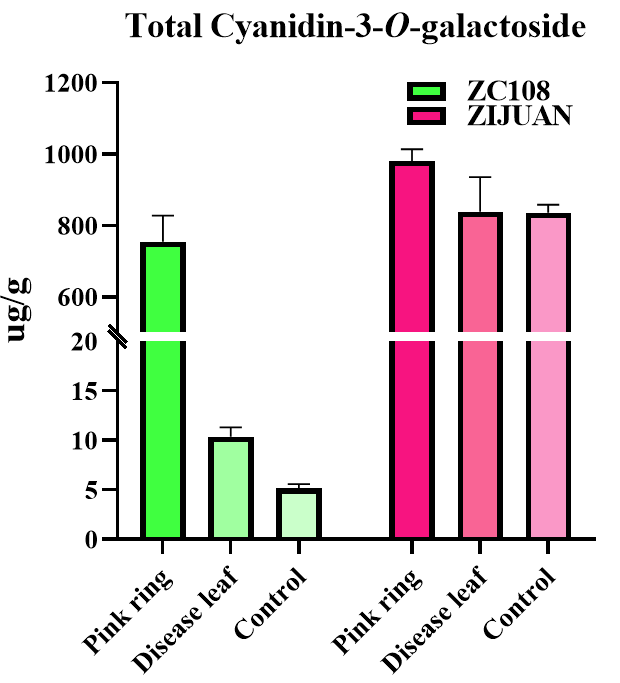


**Fig. S4** Identification of galactosylated anthocyanidin products in enzymatic reaction of recombinant UGT proteins expressed in *Escherichia coli*.

(a) Change in the ratio of transcript abundance of CsUGTs in the infected leaves to the control leaves within 6 d after infection.

(b) Phylogenetic tree of selected plants UGTs, CsUGT78A15, and CsUGT75L43 (marked with a red diamond). GenBank accession numbers of the plant GTs in this tree are CsUGT75L12, XP_028051211.1; MdUGT75B1, XP_008380456.1; AtUGT75B2, NP_172044.1; AtUGT74B1, NP_173820.1; AtUGT85A1, NP_173656.1; AtUGT87A1, NP_001323694.1; PhF3GT, Q9SBQ8.1; DcUCGalT1，AKI23632.1; CsUGT78A15，ALO19889.1;Ac-UCGalT1,ADC34700.1; UGT71G1, XP_003615613.1; AtUGT71B1,NP_188812.1; CsUGT91D1,ALO19884.1;AtUGT92A1,NP_196793.1; AtUGT89B1,NP_177529.2; AtUGT90A1,NP_179281.3; AtUGT73B1,NP_567955.1; AtF73GT, NP_567955.1; GeIF7GT, BAC78438.1; AmUGT73N1, BAG16514.1; PfUGT2, KAH6762284.1; UGT73E2, BAG16513.1.

(c) Identification of cyanidin-3*-O-*galactosides and delphinidin-3*-O-*galactoside products during enzymatic reaction of recombinant CsUGT75L43 and CsUGT78A15 proteins expressed in *Escherichia coli*.


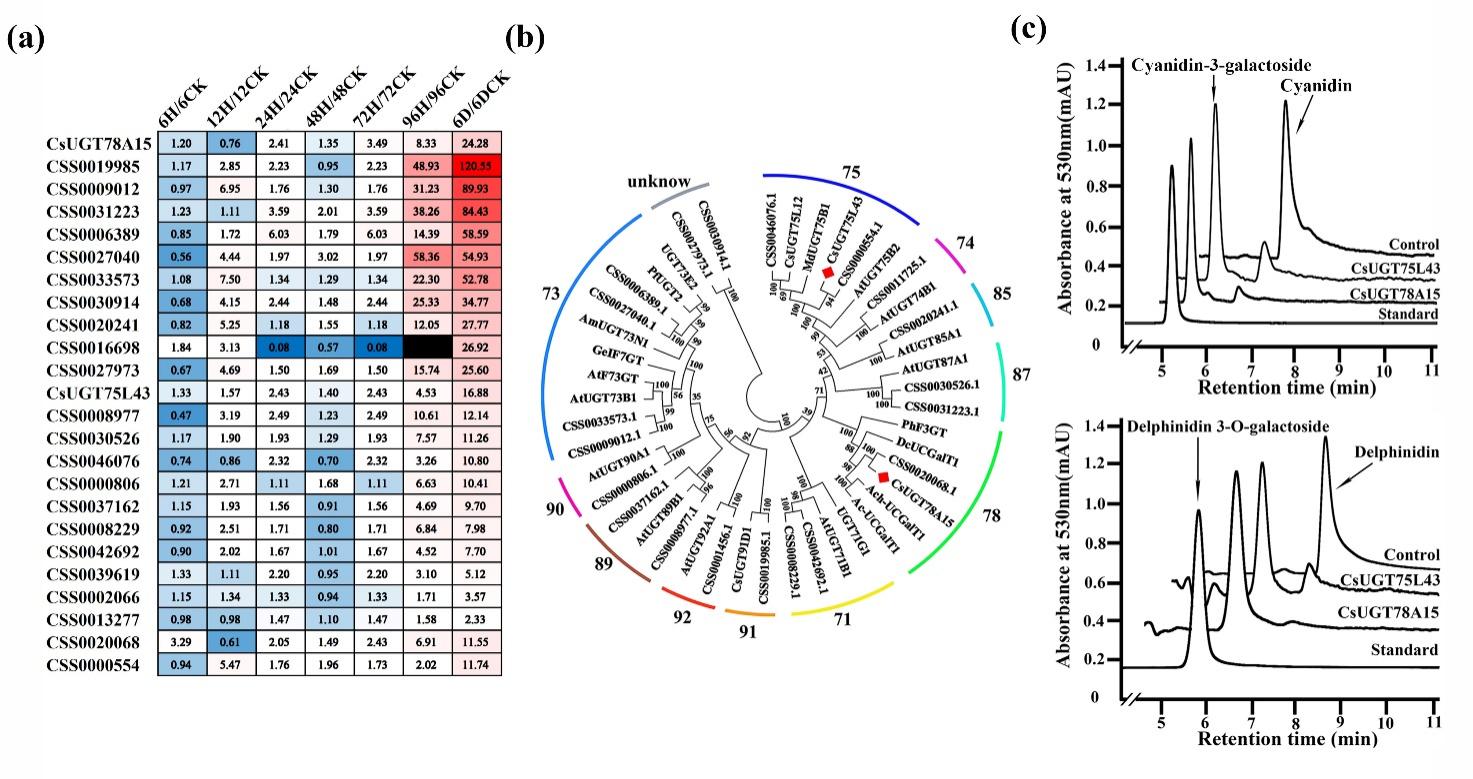


**Fig. S5** *CsMYB113* promotes the expression of the *CsANS*.


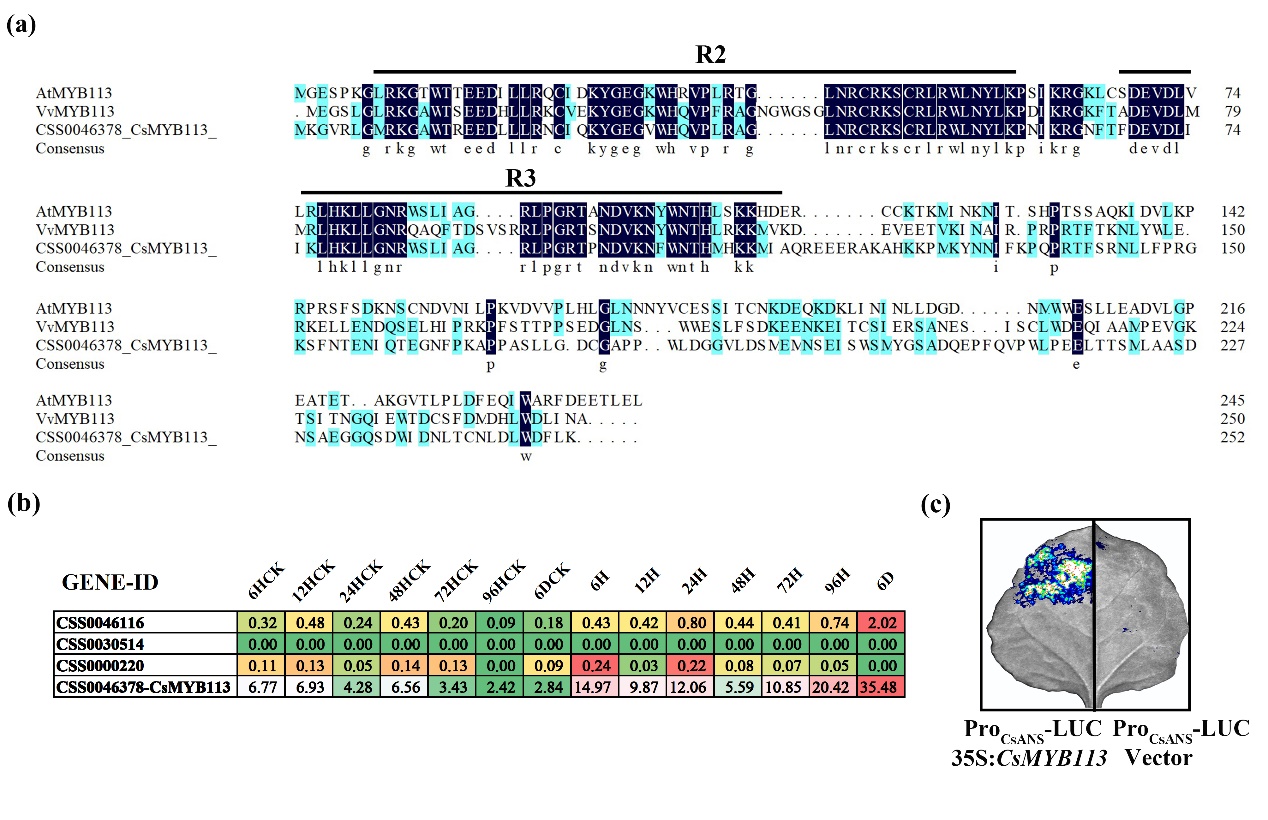
(a) *CsMYB113*, *VvMYB113*, *AtMYB113* sequence alignment.(b) Fragments per kilobase of exon model per million mapped fragments (FPKM) of CSMYB6s subfamily at different times of infection. (c) Activation of the *CsANS* promoter by *CsMYB113* in transient tobacco transformation assays.

**Fig. S6** Change in the ratio of transcript abundance of the genes of JA, SA, and ETH biosynthesis pathway and signaling pathway in the infected leaves to the control leaves within 6 d after infection. The genes include lipoxygenase (LOX), allene oxide synthase (AOS), allene oxide cyclase (AOC), 12-oxophytodienoate reductase (OPR), jasmonic acid resistant 1 (JAR1), jasmonic acid carboxyl methyltransferase (JMT), jasmonate ZIM-domain (JAZ), myelocytomatosis protein2 (MYC2), S-adenosy-L-methionine synthetase (SAMS), 1-aminocyclopropane-1-carboxylic acid synthase (ACS), 1-aminocyclopropane-1-carboxylic acid oxidase (ACO), constitutive triple response 1 (CTR), ethylene insensitive 2 (EIN2), ethylene insensitive 3/EIN3-like (EIN3/EIL), ethylene response factor (ERF), isochorismate (ICS), phenylalanine ammonia-lyase (PAL), abnormal inflorescence meristem 1 (AIM1), avrPphB susceptible 3 (PBS3), enhanced pseudomonas susceptibility 1 (EPS1), salicylic acid carboxyl methyltransferase (SAMT), SA-binding protein (SABP), non-expressor of pathogenesis-related genes 1 (NPR), TGACG motif-binding factor (TGA), Nim-interacting1 (NIMIN), pathogenesis-related (PR). Compound Abbreviations: 13-HPOT, 13(S)-hydroperoxy-octadecatrienoic acid; 12,13-EOT,12,13(S)-epoxy-octadecatrienoic acid; OPDA,12-oxophytodienoic acid; MeJA, methyl jasmonate; JA-Ile, jasmonoyl-L-isoleucine; SAM, S-adenosylmethionine; ACC,1-aminocyclopropane-1-carboxylic acid; IC-9-Glu, isochorismate-9-glutamate; MeSA, methyl salicylate.


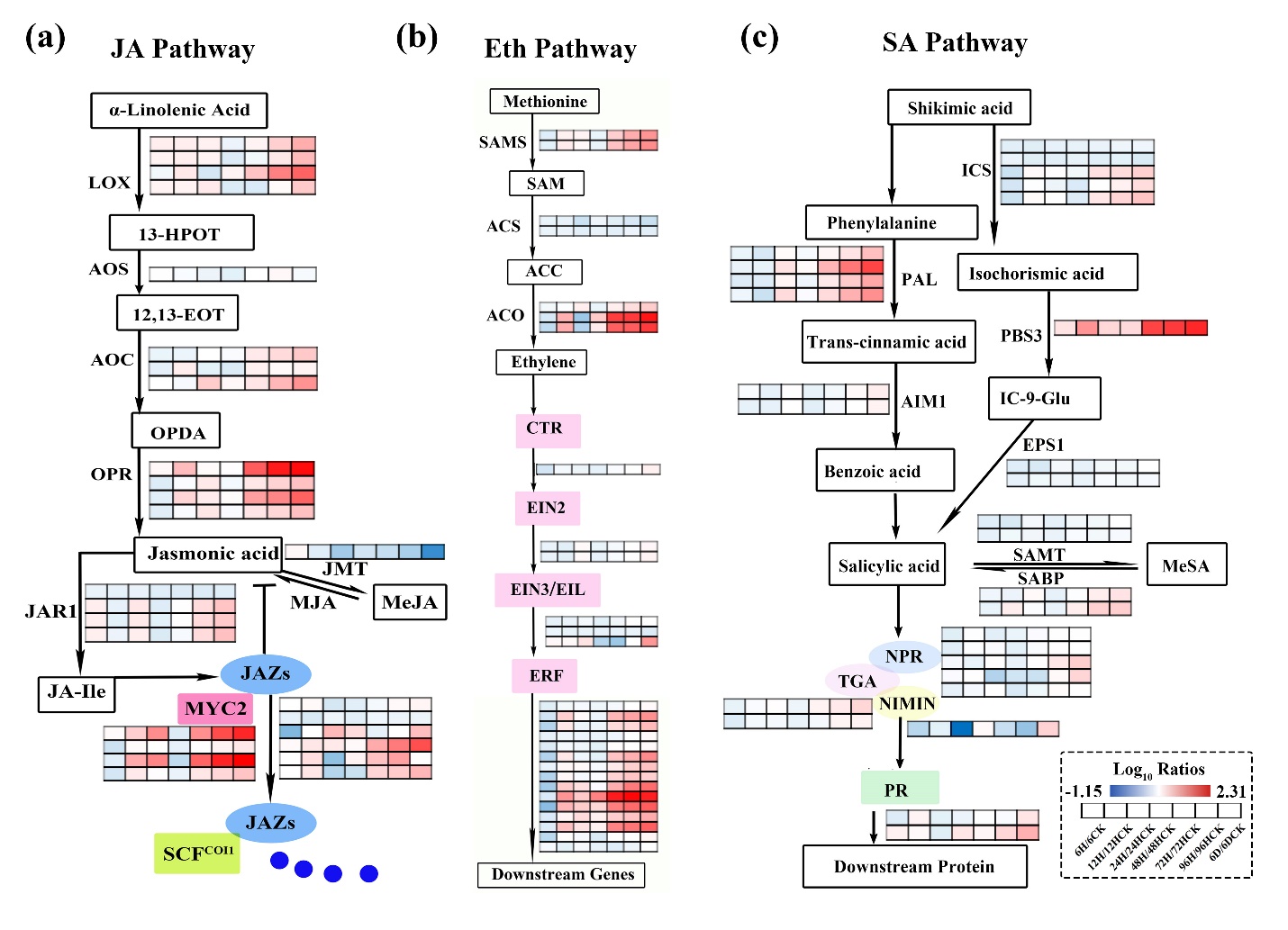


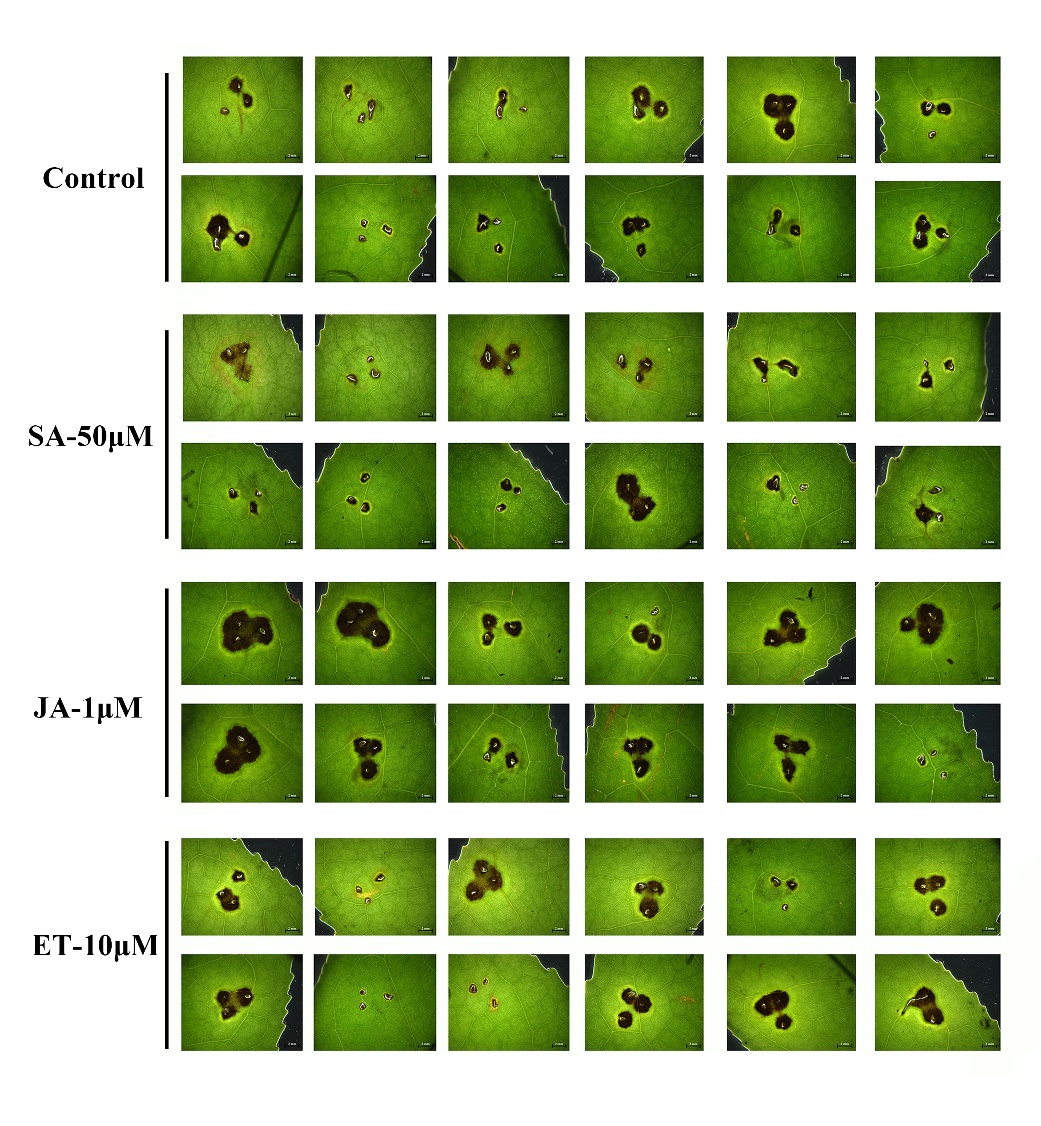
 **Fig. S7** Effects of exogenous SA, MeJA, and ETH on the symptoms of "pink ring" in annual cut seedlings after 4 dpi inoculation of cultivar LJ43. The concentration of salicylic acid is 50 μM. The concentration of jasmonic acid is 50 μM. The concentration of ethylene is 10 μM.


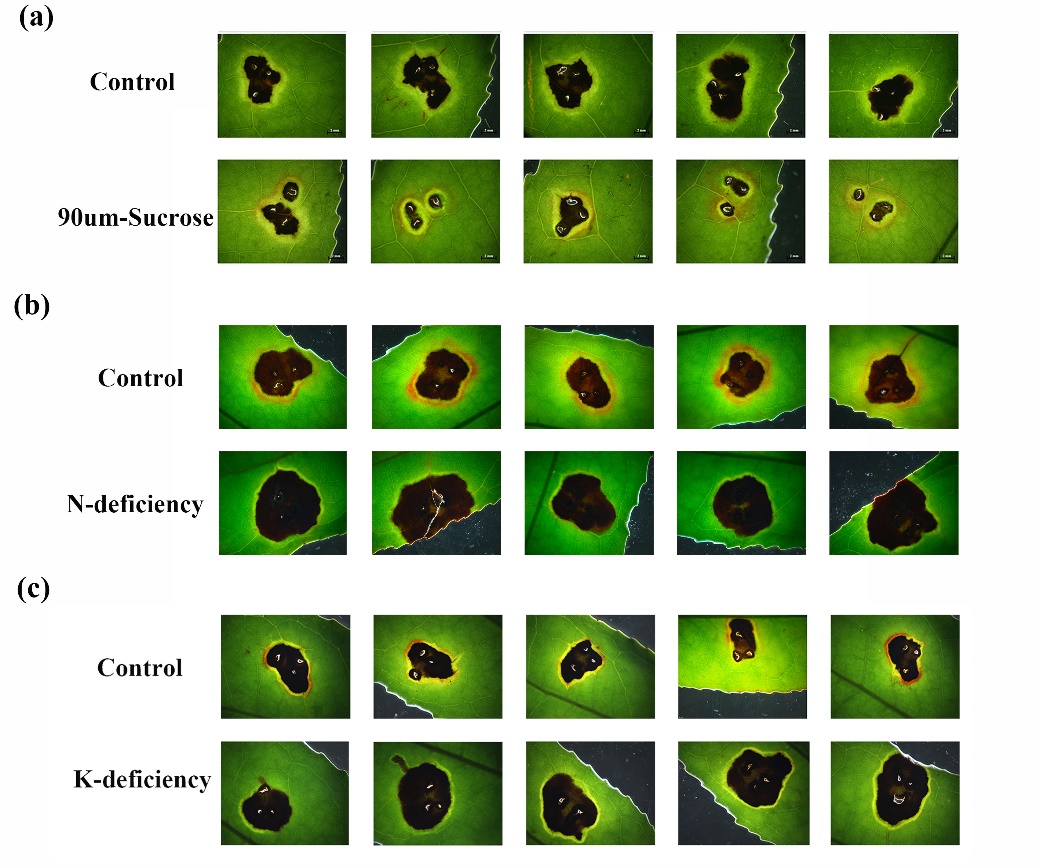
 **Fig. S8** Effects of sucrose, nitrogen (N) deficiency, and kalium (K) deficiency on “pink ring” formation and disease-resistance after pathogenic fungi *Colletotrichum* infection in tea plants. (a) Tea branches were given hydroponic treatment with 90 μM sucrose for 48 h before being inoculated with *C. camelliae*. (b), (c)Effect of nitrogen (N) and kalium (K) deficiency treatment on "pink ring" symptoms. Healthy tea seedlings were cultured in a complete nutrient solution and nitrogen (N) and kalium (K)-deficiency nutrient solution and then inoculated with *C. camelliae*.


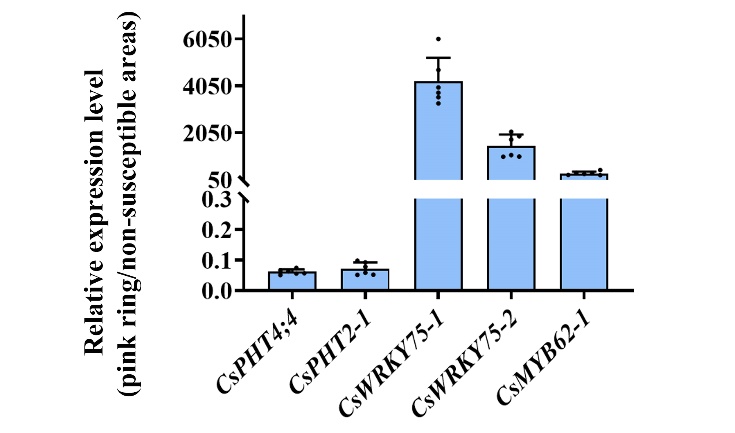
**Fig. S9** Differences in the gene expressions of the phosphate transporter protein *CsPHTs* and the Pi-deficient response genes *CsWRKY75s* and *CsMYB62-1* between “pink ring” and non-susceptible areas.


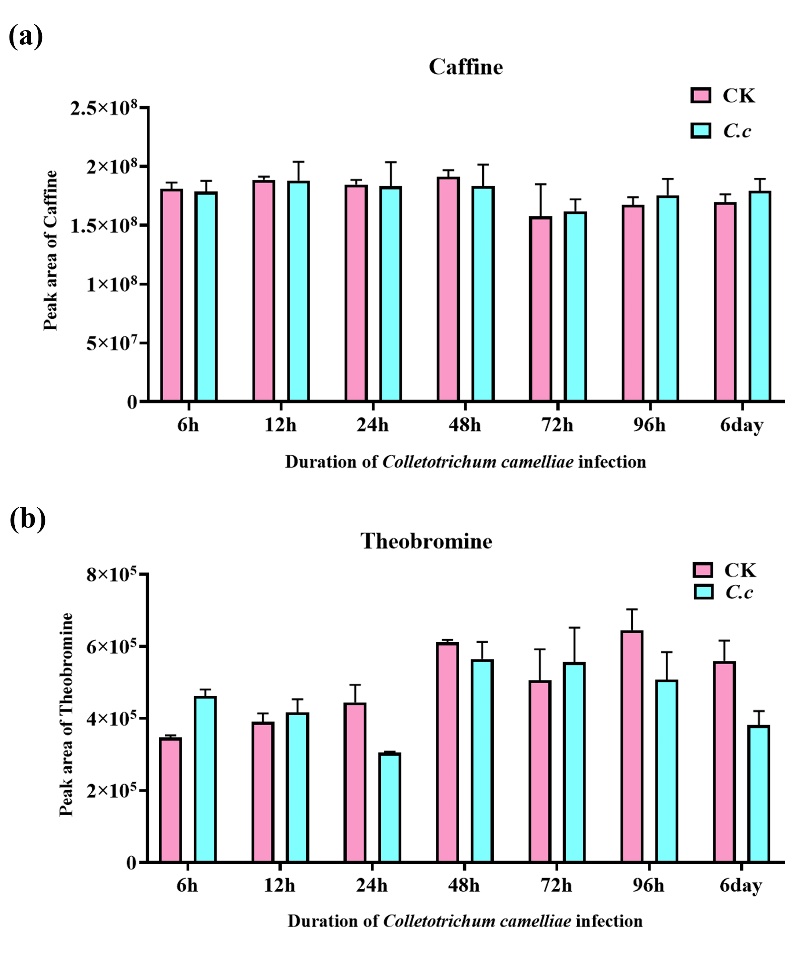
**Fig. S10** Quantification of caffeine and theobromine during anthracnose infection. (a) Peak area of caffeine duration of *C. camelliae* infection. (b) Peak area of theobromine duration of *C. camelliae* infection.

**Supplementary methods**

### **Chemical reagents**

Chemical standards cyanidin (528-58-5), cyanidin-3-galactoside (27661-36-5), delphinidin (528-53-0), and delphinidin 3-O-galactoside (197250-28-5) were purchased from Naturewill Co., Ltd. (Sichuan, China). UDP-galactose was procured from SigmaAldrich (St Louis, MO, USA). HPLC-grade methanol, acetonitrile, and acetic acid were obtained from Tedia Co., Ltd. (Fairfield, OH, USA).

### **Observation of the surface and paraffin section of infected leaves**

The symptoms of the lesion in leaves were recorded using a stereo microscope (ZEISS,Germany). The microscopic structures of the bare-handed and paraffin sections of infected leaves with *C. camelliae* were observed using an optical microscope (NIKON Eclipse, Japan). The protoplast was extracted from the “pink ring” tissues according to the procedure mentioned in a previous study^82^ and recorded using an optical microscope (NIKON Eclipse, Japan).

The fixed diseased leaves were placed into the dehydrator and dehydrated with gradient alcohol. The wax-soaked tissue was embedded in the embedding machine (Wuhan Junjie Electronics Co., Ltd., Wuhan, China). After cooling, the modified tissue chip wax block was sliced on a paraffin slicer (Shanghai Leica Instrument Co., Ltd.,Shanghai, China) at a thickness of 4 μM. The sections were dewaxed with absolute ethanol and then stained with Safranin O staining solution and plant solid green staining solution, respectively. The samples are decolorized with a series of graded ethanol after each staining. Finally, the tissue sections were mounted with neutral balsam and observed under a microscope, and the images were taken by microscope (NIKON Eclipse, Japan).

### **Scanning electron microscopy**

The morphology of hyphae and spores in infected leaves was recorded using an electron microscope. The infected leaves were first fixed with a fixative for 2 h with electron microscope fixative. The fixing procedure was as follows: first, the leaves were flushed with 0.1 M phosphate buffer (PB; pH 7.4) thrice, 15 min each time, and then fixed using 1% osmic acid solution for 1–2 h at 25 ℃ away from light. The samples were flushed again with 0.1 M PB thrice, dehydrated with a series of graded ethanol, and finally dried in a critical point dryer (Quorum, America). The pathogenic fungi *C. camelliae* in infected leaves infected were observed using a scanning electron microscope (SU8100,HITACHI, Japan).

### ***In vitro* fungicidal experiments**

Cyanidin-3*-O-*galactoside was formulated with dimethyl sulfoxide (DMSO) to prepare a concentration of 100 mg.mL^-1^ and sterilized by a 0.22 μM filter. A sterile hole punch with a diameter of 8 mm was used to dig out a piece of the fungus cake on the *C. camelliae* after 7 days of incubation. The fungus cake was placed in the center of the drug-containing medium and cultured at 28 °C. The control medium contained the same concentration of DMSO. When the area of mycelia on the control medium increased to 2/3 of the medium area, the colony radius on the treatment and CK mediums were quantified, and the mycelia morphology was recorded using an electron microscope. Three biological replicates were used in this experiment.

### ***Colletotrichum camelliae* recovery assay**

Execution of the *C. camelliae* recovery assay was performed according to the referenced literature^1^. The general procedure of the experiment is as follows: To assess the impact of *C. camelliae* infection, 4.5cm^2^ leaf fragments were collected from disease leaf after 6 days of inoculation. These stem segments were meticulously sterilized by treating them with 75% ethanol for 1 minute and then with 5% NaClO for 45 seconds. Subsequently, the sterilized fragments were rinsed thrice with sterile water. Following this, the fragments were divided into five parts, which were then placed onto potato dextrose agar plates. The plates were subsequently incubated at a temperature of 25 °C. For the sake of reliability, each experiment was conducted with three biological replicates.

### **RNA isolation and transcriptome sequencing and quantitative real-time PCR analysis**

Total RNA of leaves samples were extracted for transcriptome sequencing using an Ultra RNA Library Prep Kit (NEB#7530, New England Biolabs). The cDNA/DNA/small RNA libraries were sequenced on the Illumina HiSeq6000 sequencing platform by Genedenovo Biotechnology Co., Ltd. (Guangzhou, China). The differentially expressed genes were mapped to each term of the GENEONTOLOGY(GO) database (http://www.geneontology.org), and the number of genes for each term was calculated to obtain a list of genes with a certain GO function and the number of genes.

The total RNA of leaf samples for quantitative real-time PCR analysis (qRT-PCR) was extracted using the RNAprep Pure kit (Tiangen, Beijing, China). A total of 1 μg of RNA samples were converted into first-stranded cDNAs using the PrimeScrip RT enzyme (Takara, Japan). The qRT-PCR was performed using Hieff qPCR SYBR Green Master Mix (No-Rox) (YEASEN, Shanghai, China) on the CFX 96 ^TM^ System (Bio-Rad, Hercules, CA, USA). The reaction procedure used a temperature of 95 °C for 5 min; 40 cycles, 10 sec at 95 °C, and then 30 sec at 58 °C. The data results were calculated using the 2^–ΔΔct^ method to calculate the relative expression. The glyceraldehyde-3-phosphate dehydrogenase (GAPDH) gene was used as an internal reference gene. The gene-specific primer sequences for qRT-PCR analysis are listed in Table S7.

### **Expression and product identification of recombinant CsUGT proteins**

The full-length ORFs of *CsUGT78A15* and *CsUGT75L43* were cloned into the vector pMAL-c2x (Novagen, Schwalbach, Germany) and then introduced into *Escherichia coli* BL21 (DE3) (Transgen, Beijing, China). Subsequently, 0.1 mM isopropyl b-D-thiogalactoside was used to induce the expression of recombinant proteins from positive colonies at 16 °C for 24 h. Maltose binding protein fusion proteins expressed in *E. coli* were purified using amylose resin affinity chromatography and analyzed by 12% sodium dodecyl sulphate–polyacrylamide gel electrophoresis (SDS-PAGE).

To analyze the in vitro activity of purified recombinant CsUGT proteins, the enzymatic reaction was performed at 30 °C for 30 min in 50 μL of reaction solution consisting of 100 mM Tris-HCl buffer (pH 7), 2.5 mM UDP-Gal as sugar donor, 0.5 mM substrate (cyanidin, delphinidin) as sugar receptor, and 5 μg of recombinant proteins. The control sample contained the protein purified from the sample expressing the empty vector. The enzyme reaction was terminated with 5% hydrochloric acid. The enzyme reaction products were identified using UPLC-MRM-MS/MS analysis mentioned above.

### **Exogenous hormone treatments，inorganic phosphates (Pi) deficiency test and long-term treatment of slow-release fertilizers with different N/K/P ratios**

The healthy two-year-old plants were selected for the exogenous hormone treatment before the inoculation test. The exogenous hormones included 1 μM methyl jasmonate (MeJA), 50 μM SA, and 10 μM ET. Water was the control solution. After 12 h of hormone spraying treatment, tea leaves were inoculated with *C. camelliae*.

Pi deficiency solutions were designed with a complete nutrient solution without Pi, containing 30% Pi and 60% Pi, respectively. The complete nutrient solution containing large amounts of elements and trace elements was the control solution. The healthy two-year-old tea seedlings were cultured in different nutrient solutions for 3 d and then inoculated according to the *C. camelliae* inoculation method mentioned above. At least three biological replicates of each treatment were carried out.

Three npk ratio slow-release fertilizers were mixed into the nutrient soil of tea tree cuttings. Cultivate ZC108 cuttings in the same environment for 2 years. Subsequent inoculation with pathogenic *C. camelliae*.

### **Dual LUC assay**

The dual-luciferase promoter assay was performed following the protocol described previously. Briefly, the promoter sequences for CsANS were inserted into the cloning site of pGreen 0800-LUC, while the coding sequence of the effector CsMYB113 was cloned into the vector pCAMBIA1305 and introduced into *Agrobacterium tumefaciens* strain GV3101 (pSoup19). 35s: CsMYB113-GFP was co-injected into *N. benthamiana* leaves with the promoter, and the empty vector and promoter were co-injected on the other side of the leaf as a control. Image acquisition was performed using a chemiluminescence/fluorescence image analysis system (Tanon,5200,Shanghai,China) after 60 h of normal incubation.

1. Song, Y. *et al.* BIN2 negatively regulates plant defence against Verticillium dahliae in Arabidopsis and cotton. *Plant Biotechnology Journal* **19**, 2097-2112 <http://dx.doi.org/10.1111/pbi.13640> (2021).
